# Implications of the Higgs discovery for the MSSM

**DOI:** 10.1140/epjc/s10052-013-2704-3

**Published:** 2014-05-27

**Authors:** Abdelhak Djouadi

**Affiliations:** 1 Laboratoire de Physique Théorique, U. Paris–Sud and CNRS, 91405 Orsay, France; 2 Present Address: TH Unit, CERN, Geneva, Switzerland

## Abstract

The implications of the discovery of the Higgs boson at the LHC with a mass of approximately 125 GeV are summarised in the context of the minimal supersymmetric extension of the Standard Model, the MSSM. Discussed are the implications from the measured mass and production/decay rates of the observed particle and from the constraints in the search for the heavier Higgs states at the LHC.

## Introduction

The historical discovery by ATLAS and CMS of a particle with a mass of approximately 125 GeV [1, 2] and properties that are compatible with those of a scalar Higgs boson [3–8] has far reaching consequences not only for the Standard Model (SM) of the electroweak and strong interactions, but also for new physics models beyond it. This is particularly true for supersymmetric theories (SUSY) [9–11] that are widely considered to be the most attractive extensions of the SM as they naturally protect the Higgs mass against large radiative corrections and stabilise the hierarchy between the electroweak and Planck scales, besides of allowing for the unification of the three gauge coupling constants and providing a good candidate for the dark matter in the universe, the lightest SUSY particle.

In the minimal supersymmetric extension of the SM (MSSM), two Higgs doublet fields $H_u$ and $H_d$ are required to break the electroweak symmetry, leading to a physical spectrum with five Higgs particles: two CP-even, $h$ and $H$, a CP-odd, $A$, and two charged, $H^\pm$, states [6, 8]. Two parameters are needed to describe the MSSM Higgs sector at the tree level: one Higgs mass, which is generally taken to be that of the pseudoscalar boson $M_A$, and the ratio of vacuum expectation values of the two Higgs fields, $\tan \beta =v_d/v_u$, expected to lie in the range $1 \lesssim \tan \beta \lesssim 60$. The masses of the CP-even $h,H$ and the charged $H^\pm$ states, as well as the mixing angle $\alpha$ in the CP-even sector are uniquely defined in terms of these two inputs at tree level, but this nice property is spoiled at higher orders [12–28].

At high $M_A$ values, $M_A \gg M_Z$, one is in the so-called decoupling regime [29] in which the neutral CP-even state $h$ is light and has almost exactly the properties of the SM Higgs boson, i.e. its couplings to fermions and gauge bosons are the same as the standard Higgs, while the other CP-even $H$ and the charged $H^\pm$ bosons become heavy and mass degenerate with the $A$ state, $M_H \approx M_{H^\pm } \approx M_A$, and they decouple from the massive gauge bosons. In this regime, the MSSM Higgs sector thus looks almost exactly like the one of the SM with its unique Higgs boson.

There is, however, one major difference between the two cases: while in the SM the Higgs mass is essentially a free parameter (and should simply be smaller than about 1 TeV in order to ensure unitarity in the high-energy scattering of massive gauge bosons), the lightest MSSM CP-even Higgs particle mass is bounded from above and, depending on the SUSY parameters that enter the important quantum corrections, is restricted to $M_h^\mathrm{max} \approx 90$–130 GeV. The lower value comes from experimental constraints, in particular Higgs searches at LEP [30, 31], while the upper bound assumes a SUSY-breaking scale that is not too high, $M_S \lesssim {\mathcal {O}}$ (1 TeV), in order to avoid too much fine-tuning in the model. Hence, the requirement that the MSSM $h$ boson coincides with the one observed at the LHC, i.e. with $M_h \approx 125$ GeV and almost SM-like couplings as the LHC data seem to indicate, would place very strong constraints on the MSSM parameters, in particular the SUSY scale $M_S$, through their contributions to the radiative corrections to the Higgs sector. This comes in addition to the limits that have been obtained from the search of the heavier Higgs states at the LHC, as well as from the negative search for supersymmetric particles.

In this review, we summarise the implications of the available LHC Higgs results for the MSSM Higgs sector. We first discuss the consequences of the $M_h$ measured value for the various unconstrained (with the many free parameters defined at the weak scale) and constrained (with parameters obeying some universal boundary conditions at the high scale) versions of the MSSM. We then discuss the impact of the measured production and decay rates of the observed particle on the various Higgs couplings and, hence, the MSSM parameters. The impact of the negative search of the heavy $H,A$ and $H^\pm$ states is summarised. An outlook is given in a concluding section.

## Implications of the Higgs mass value

### The Higgs masses in the MSSM

In the MSSM, the tree-level masses of the CP-even $h$ and $H$ bosons depend only on $M_A$ and $\tan \beta$. However, many parameters of the MSSM such as the masses of the third generation stop and sbottom squarks $m_{{\tilde{t}}_i}, m_{{\tilde{b}}_i}$ and their trilinear couplings $A_{t}, A_b$ enter $M_h$ and $M_H$ through quantum corrections. In the basis $(H_d,H_u)$, the CP-even Higgs mass matrix can be written in full generality as

1 $$\begin{aligned} {\mathcal {M}}^2&= M_{Z}^2 \left( \begin{array}{c@{\quad }c} c^2_\beta &{} -s_\beta c_\beta \\ -s_\beta c_\beta &{} s^2_\beta \\ \end{array}\right) +M_{A}^2 \left( \begin{array}{c@{\quad }c} s^2_\beta &{} -s_\beta c_\beta \\ -s_\beta c_\beta &{} c^2_\beta \\ \end{array} \right) \nonumber \\&+ \left( \begin{array}{c@{\quad }c} \varDelta {\mathcal {M}}_{11}^2 &{} \varDelta {\mathcal {M}}_{12}^2 \\ \varDelta {\mathcal {M}}_{12}^2 &{}\varDelta {\mathcal {M}}_{22}^2 \\ \end{array}\right) \end{aligned}$$

where we use the short-hand notation $s_\beta \equiv \sin \beta$ etc. and introduce the radiative corrections by a general $2\times 2$ matrix $\varDelta {\mathcal {M}}_{ij}^2$. One can then easily derive the neutral CP even Higgs boson masses and the mixing angle $\alpha$ that diagonalises the $h$ and $H$ states, $H= \cos \alpha H_d^0 + \sin \alpha H_u^0$ and $h=-\sin \alpha H_d^0 + \cos \alpha H_u^0$:

2 $$\begin{aligned}&M_{h/H}^2=\frac{1}{2} \big ( M_{A}^2+M_{Z}^2+ \varDelta {\mathcal {M}}_{11}^2+ \varDelta {\mathcal {M}}_{22}^2 \mp N \big ) \end{aligned}$$

3 $$\begin{aligned}&\tan \alpha =\frac{2\varDelta {\mathcal {M}}_{12}^2 - (M_{A}^2 + M_{Z}^2) s_{\beta }}{ \varDelta {\mathcal {M}}_{11}^2 - \varDelta {\mathcal {M}}_{22}^2 + (M_{Z}^2-M_{A}^2) c_{2\beta } + N }\end{aligned}$$

4 $$\begin{aligned}&N = \sqrt{M_{A}^4 + M_{Z}^4 - 2 M_{A}^2 M_{Z}^2 c_{4\beta } + C} \nonumber \\&C = 4 \varDelta {\mathcal {M}}_{12}^4 + ( \varDelta {\mathcal {M}}_{11}^2 - \varDelta {\mathcal {M}}_{22}^2)^2 - 2 (M_{A}^2 - M_{Z}^2)\nonumber \\&\qquad \quad \times ( \varDelta {\mathcal {M}}_{11}^2 - \varDelta M_{22}^2) c_{2\beta } - 4 (M_{A}^2 + M_{Z}^2) \varDelta {\mathcal {M}}_{12}^2 s_{2\beta } \nonumber \\ \end{aligned}$$

The by far leading one-loop radiative corrections to the mass matrix of Eq. (1) are controlled by the top Yukawa coupling, $\lambda _t = m_t/v \sin \beta$ with $v=246$ GeV, which appears with the fourth power. One obtains a very simple analytical expression for the radiative correction matrix $\varDelta {\mathcal {M}}_{ij}^2$ if only this contribution is taken into account [12–14]

5 $$\begin{aligned} \varDelta {\mathcal {M}}_{11}^2&\sim \varDelta {\mathcal {M}}_{12}^2 \sim 0 \ , \\ \varDelta {\mathcal {M}}_{22}^2&\sim \epsilon = \frac{3 \bar{m}_t^4}{2\pi ^2 v^2\sin ^ 2\beta } \left[ \log \frac{M_S^2}{\bar{m}_t^2} \!+\! \frac{X_t^2}{M_S^2} \!\left( 1 \!-\! \frac{X_t^2}{12M_S^2} \right) \right] \nonumber \end{aligned}$$

where $M_S$ is the geometric average of the two stop masses $M_S =\sqrt{ m_{\tilde{t}_1}m_{\tilde{t}_2}}$ defined to be the SUSY-breaking scale and $X_{t}$ is the stop mixing parameter given by $X_t = A_t - \mu /\tan \beta$ with $\mu$ the higgsino mass parameter; $\bar{m}_t$ is the running $\mathrm{\overline{MS}}$ top quark mass to account for the leading two-loop QCD corrections in a renormalisation-group improved approach (some refinements can be include as well).

Other soft SUSY-breaking parameters, in particular $\mu$ and $A_b$ (and in general the corrections controlled by the bottom Yukawa coupling $\lambda _b = m_b/v \cos \beta$, which at large value of $\mu \tan \beta$ become relevant) as well as the gaugino mass parameters $M_{1,2,3}$, provide a small but non-negligible correction to $\varDelta {\mathcal {M}}_{ij}^2$ and can thus also have an impact on the loop corrections [15, 16, 25–28].

The maximal value of the $h$ mass, $M_h^\mathrm{max}$ is given in the leading one–loop approximation above by

6 $$\begin{aligned} M_h^2 \mathop {\rightarrow }\limits ^{M_A \gg M_Z}M_Z^2 \cos ^2 2 \beta + \varDelta {\mathcal {M}}_{22}^2 \end{aligned}$$

and is obtained for the choice of parameters [25–28]:

- a decoupling regime with heavy $A$ states, $M_A \sim {\mathcal {O}}$(TeV);
- large values of the parameter $\tan \beta$, $\tan \beta \gtrsim 10$;
- heavy stops, i.e. large $M_S$ values and we choose in general $M_S \le 3$ TeV to avoid a too large fine-tuning [32, 33];
- a stop trilinear coupling $X_t=\sqrt{6}M_S$, the so-called maximal mixing scenario that maximises the stop loops [34].

**Fig. 1:**
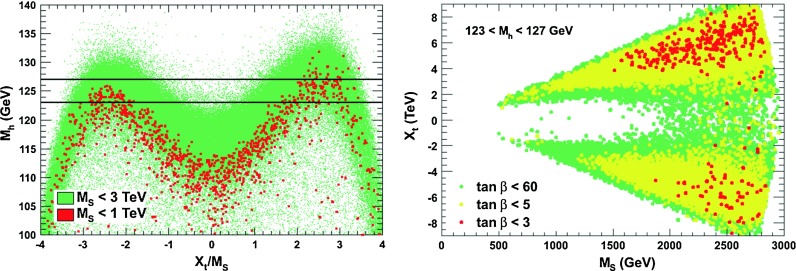
The maximal value of the $h$ boson mass as a function of $X_t/M_S$ in the pMSSM when all other soft SUSY-breaking parameters and $\tan \beta$ are scanned (*left*) and the contours for the Higgs mass range 123 $<M_h<$ 127 GeV in the $[M_S,X_t]$ plane for some selected range of $\tan \beta$ values (*right*); from Ref. [35]

If the parameters are optimised as above, the maximal $h$ mass value can reach the level of $M_h^\mathrm{max} \approx 130$ GeV.

An important aspect is that in the decoupling regime $M_A \gg M_Z$, the heavier CP-even and the charged Higgs states become almost degenerate in mass with the CP-odd state, $M_H \approx M_{H^\pm } \approx M_A$, while the mixing angle $\alpha$ becomes close to $\alpha \approx \frac{\pi }{2} -\beta$ making the couplings of the light $h$ state to fermions and massive gauge bosons SM-like, and decoupling the $H,H^\pm$ from the weak bosons as is the case for the state $A$ by virtue of CP invariance.

In this section, we discuss the implications of the measured mass value of the observed Higgs boson at the LHC [35–62] that we identify with the lightest state $h$ of the MSSM. We consider the phenomenological MSSM [63] in which the relevant soft SUSY parameters are allowed to vary freely (but with some restrictions) and constrained MSSM scenarios such as the minimal supergravity (mSUGRA) [64–67], gauge mediated (GMSB) [68–72] and anomaly mediated (AMSB) [73–75] supersymmetry-breaking models (for a review, see again Ref. [8]). We also discuss the implications of such an $M_h$ value for scenarios in which the supersymmetric spectrum is extremely heavy, the so-called split SUSY [76–78] or high-scale SUSY models [79, 80]. Finally, a new parametrisation of the Higgs sector, which uses the crucial information $M_h = 125$ GeV, is discussed [81].

### Implications for the phenomenological MSSM

In an unconstrained MSSM, there is a large number of soft SUSY-breaking parameters, ${\mathcal {O}}(100)$, but analyses can be performed in the so-called “phenomenological MSSM” (pMSSM) [63], in which CP conservation, flavour diagonal sfermion mass and coupling matrices and universality of the first and second sfermion generations are imposed. The pMSSM involves then 22 free parameters in addition to those of the SM: besides $\tan \beta$ and $M_A$, these are the higgsino mass $\mu$, the three gaugino masses $M_{1,2,3}$, the diagonal left- and right-handed sfermion mass parameters $m_{ {\tilde{f}}_{L,R}}$ and the trilinear sfermion couplings $A_f$.

As discussed above, an estimate of the upper bound on $M_h$ can be obtained by including the corrections that involve only the parameters $M_S$ and $X_t$. However, to be more precise, one could scan the full pMSSM 22 parameter space in order to include the subleading corrections. To do so, one can use RGE programs such as Suspect [82] which calculate the Higgs and superparticle spectrum in the MSSM including the most up-to-date information [25].

To obtain the value $M_h^\mathrm{max}$ with the full radiative corrections, a large scan of the pMSSM parameters in an uncorrelated way was performed [35, 36] in the domains:

7 $$\begin{aligned} 1\le \tan \beta \le 60, \ 50\,\mathrm{GeV} \le M_A \le 3\,\mathrm{TeV}, \nonumber \\ -9\,\mathrm{TeV} \le A_t, A_b, A_\tau \le 9\,\mathrm{TeV},\nonumber \\ 50\,\mathrm{GeV} \le m_{\tilde{f}_L}, m_{\tilde{f}_R}, M_3 \le 3\,\mathrm{TeV}, \nonumber \\ 50\,\mathrm{GeV} \le M_1, M_2, |\mu | \le 1.5\,\mathrm{TeV}. \end{aligned}$$

The results are shown in Fig. 1, where, in the left-hand side, the obtained maximal value $M_h^\mathrm{max}$ is displayed as a function of the ratio of parameters $X_t/M_S$. The resulting values are confronted to the mass range $123\,\mathrm{GeV} \le M_h \le 127\,\mathrm{GeV}$ when the parametric uncertainties from the SM inputs such as the top quark mass and the theoretical uncertainties in the determination of $M_h$ are included^1^.

For $M_S \lesssim 1$ TeV, only the scenarios with $X_t/M_S$ values close to maximal mixing $X_t/M_S \approx \sqrt{6}$ survive. The no-mixing scenario $X_t \approx 0$ is ruled out for $M_S \lesssim 3$ TeV, while the typical mixing scenario, $X_t \approx M_S$, needs large $M_S$ and moderate to large $\tan \beta$ values. From the scan, one obtains a maximum $M_h^\mathrm{max}=136$, 126 and 123 GeV with maximal, typical and zero mixing, respectively.

What are the implications for the mass of the lightest stop state $\tilde{t}_1$? This is illustrated in the right-hand side of Fig. 1, where the contours are shown in the $[M_S,X_t]$ plane in which one obtains $123 < M_h <127$ GeV from the pMSSM scan; the regions in which $\tan \beta \lesssim 3, 5$ and 60 are highlighted. One sees again that a large part of the parameter space is excluded if the Higgs mass constraint is imposed. In particular, large $M_S$ values, in general corresponding to large $m_{{\tilde{t}}_1}$ are favoured. However, as $M_S = \sqrt{ m_{{\tilde{t}}_1} m_{{\tilde{t}}_2}}$, the possibility that $m_{{\tilde{t}}_1}$ is of the order of a few 100 GeV is still allowed, provided that stop mixing (leading to a significant $m_{{\tilde{t}}_1}, m_{{\tilde{t}}_2}$ splitting) is large [36, 57–59].

**Fig. 2:**
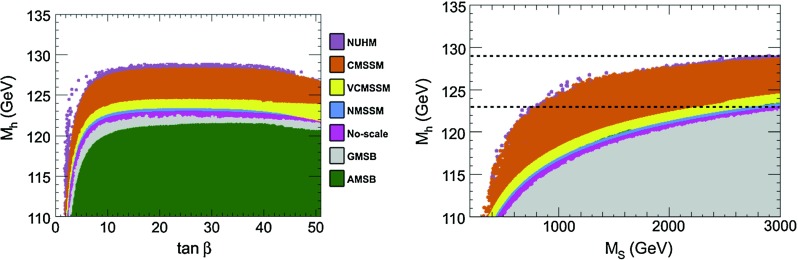
The maximal value of the $h$ boson mass as a function of $\tan \beta$ (*left*) and $M_S$ (*right*) with a scan of all other parameters in various constrained MSSM scenarios. The range $123 < M_h < 129$ GeV for the light $h$ boson mass is highlighted. From Ref. [35]

Masses above 1 TeV for the scalar partners of light quarks and for the gluinos are also required by the direct searches of SUSY particles at the LHC [86, 87], confirming the need of high $M_S$ values. Nevertheless, relatively light stops as well as electroweak sparticles such as sleptons, charginos and neutralinos are still possible allowing for a “natural SUSY” [33] in spite of the value $M_h \approx 125$ GeV. Nevertheless, the present LHC SUSY searches [86, 87] are constraining more and more this natural scenario.

### Implications for constrained MSSM scenarios

In constrained MSSM scenarios (cMSSM), the various soft SUSY-breaking parameters obey a number of universal boundary conditions at a high energy scale, thus reducing the number of basic input parameters to a handful. The various soft SUSY-breaking parameters are evolved via the MSSM renormalisation group equations down to the low energy scale $M_S$ where the conditions of proper electroweak symmetry breaking (EWSB) are imposed.

Three classes of such models have been widely discussed in the literature. There is first the minimal supergravity (mSUGRA) model [64–67] in which SUSY-breaking is assumed to occur in a hidden sector which communicates with the visible sector only via flavour-blind gravitational interactions, leading to universal soft breaking terms, namely a common $m_{1/2}, m_0, A_0$ values for the gaugino masses, sfermion masses and sfermion trilinear couplings. Then come the gauge mediated [68–72] and anomaly mediated [73–75] SUSY-breaking (GMSB and AMSB) scenarios in which SUSY-breaking is communicated to the visible sector via, respectively, gauge interactions and a super-Weyl anomaly.

These models are described by $\tan \beta$, the sign of $\mu$ and a few continuous parameters. Besides of allowing for both signs of $\mu$, requiring $1\le \tan \beta \le 60$ and, to avoid excessive fine-tuning in the EWSB conditions, imposing the bound $M_S =M_\mathrm{EWSB} < 3 \mathrm{TeV}$, we adopt the following ranges for the input parameters of these scenarios:

**Table:** 

mSUGRA	50 GeV $\le m_0 \le$ 3 TeV	50 GeV $\le m_{1/2} \le$ 3 TeV	$|A_0|\le 9$ TeV
GMSB	10 TeV $\le \varLambda \le$ 1000 TeV	1 $\le M_\mathrm{mess} / \varLambda \le 10^{11}$	$N_\mathrm{mess} =$ 1
AMSB	1 TeV $\le m_{3/2}\le$ 100 TeV	50 GeV $\le m_0\le$ 3 TeV	

Hence, in contrast to the pMSSM, the various parameters which enter the radiative corrections to $M_h$ are not all independent in these constrained scenarios, as a consequence of the relations between SUSY-breaking parameters that are set at the high-energy scale and the requirement that electroweak symmetry breaking is triggered radiatively for each set of input parameters. The additional constraints make that it is not possible to freely tune the parameters that enter the Higgs sector to obtain the pMSSM maximal value of $M_h$. In order to obtain even a rough determination of $M_h^\mathrm{max}$ in a given constrained SUSY scenario, it is necessary to scan through the allowed range of values for the basic input parameters.

Using again the program Suspect, a full scan of these scenarios has been performed in Ref. [35] and the results for $M_h^\mathrm{max}$ are shown in the left-hand side of Fig. 2 as a function of $\tan \beta$, the input parameter that is common to all models, and in the right-hand side of the figure as a function of $M_S$. In the adopted parameter space of the models and with the central values of the SM inputs, the obtained upper $h$ mass value is $M_h^\mathrm{max} \approx$121 GeV in the AMSB scenario, i.e. much less that 125 GeV, while in the GMSB scenario one has $M_h^\mathrm{max} \approx$122 GeV (these values are obtained for $\tan \beta \approx 20$). Thus, clearly, these two scenarios are disfavoured if the lightest $h$ particle has indeed a mass in the range 123–127 GeV and $M_S \lesssim 3$ TeV. In mSUGRA, one obtains $M_h^\mathrm{max} = 128$ GeV and, thus, some parameter space would still survive the $M_h$ constraint.

The upper bound on $M_h$ in these scenarios can be qualitatively understood by considering in each model the allowed values of the trilinear coupling $A_t$, which essentially determines the stop mixing parameter $X_t$ and thus the value of $M_h$ for a given scale $M_S$. In GMSB, one has $A_t\approx 0$ at relatively high scales and its magnitude does not significantly increase in the evolution down to the scale $M_S$; this implies that we are almost in the no-mixing scenario which gives a low value of $M_h^\mathrm{max}$ as can be seen from Fig. 1. In AMSB, one has a non-zero $A_t$ that is fully predicted at any renormalisation scale in terms of the Yukawa and gauge couplings; however, the ratio $A_t/M_S$ with $M_S$ determined from the overall SUSY breaking scale $m_{3/2}$ turns out to be rather small, implying again that we are close to the no-mixing scenario. Finally, in the mSUGRA model, since we have allowed $A_t$ to vary in a wide range as $|A_0|\le 9$ TeV, one can get a large $A_t/M_S$ ratio, which leads to a heavier Higgs particle. However, one cannot easily reach $A_t$ values such that $X_t/M_S \approx \sqrt{6}$ so that we are not in the maximal-mixing scenario and the higher upper bound on $M_h$ in the pMSSM cannot be reached.

In the case of mSUGRA, one can study several interesting special cases: the no-scale scenario with $m_0 \approx A_0 \approx 0$ [88, 89], the scenario $m_0 \approx 0$ and $A_0 \approx - \frac{1}{4} m_{1/2}$, which approximately corresponds to the constrained next-to-MSSM (cNMSSM) [90, 91], $A_0 \approx -m_0$, which corresponds to a very constrained MSSM (VCMSSM) [92], and a non-universal Higgs mass model (NUHM) [93] in which the soft SUSY-breaking scalar mass terms are different for the sfermions and for the two Higgs doublet fields.

In two particular cases, namely the “no-scale” and the “approximate cNMSSM” scenarios, the upper bound on $M_h$ is much lower than in the more general mSUGRA case and, in fact, barely reaches $M_h \approx 123$ GeV. The main reason is that these scenarios involve small values of $A_0$ at the GUT scale, $A_0 \approx 0$ for no-scale and $A_0 \approx -\frac{1}{4} m_{1/2}$ for the cNMSSM, which lead to $A_t$ values at the weak scale that are too low to generate a significant stop mixing and, hence, one is again close to the no-mixing scenario. Thus, only a very small fraction of the parameter space of these two sub-classes of the mSUGRA model survive if we impose 123 $< M_h <$ 127 GeV. These models should thus have a very heavy sfermion spectrum as a value $M_S \gtrsim 3$ TeV is required to increase $M_h^\mathrm{max}$. In the VCMSSM case, the value $M_h \simeq 125$ GeV can be reached as $|A_0|$ can be large for large $m_0$, $A_0 \approx -m_0$, allowing for typical mixing.

Finally, since the NUHM is more general than mSUGRA as we have two more free parameters, the $[\tan \beta , M_h]$ area shown in Fig. 2 is larger than in mSUGRA. However, since we are in the decoupling regime and the value of $M_A$ does not matter much (as long as it a larger than a few hundred GeV) and the key weak-scale parameters entering the determination of $M_h$, i.e. $\tan \beta , M_S$ and $A_t$ are approximately the same in both models, one obtains a bound $M_h^\mathrm{max}$ that is only slightly higher in NUHM compared to the mSUGRA case.

In these constrained scenarios and, in particular in the general mSUGRA model, most of the scanned points giving the appropriate Higgs mass correspond to the decoupling regime of the MSSM Higgs sector and, hence, to an $h$ boson with a SM–Higgs cross section and branching ratios. Furthermore, as the resulting SUSY spectrum for $M_h = 125 \pm 2$ GeV is rather heavy in these scenarios (easily evading the LHC limits from direct sparticle searches [86]), one obtains very small contributions to observables like the anomalous muon magnetic moment $(g-2)_\mu$ and to $B$-physics observables such as the rates BR($B_s \rightarrow \mu ^+\mu ^-$) or BR$(b \rightarrow s\gamma )$ [94]. Hence, the resulting spectrum complies with all currently available constraints. In addition, as will be discussed later, the correct cosmological density for the LSP neutralino required by recent measurements [95] can easily be satisfied. The $M_h$ value provides thus a unique constraint in this decoupling regime.

**Fig. 3:**
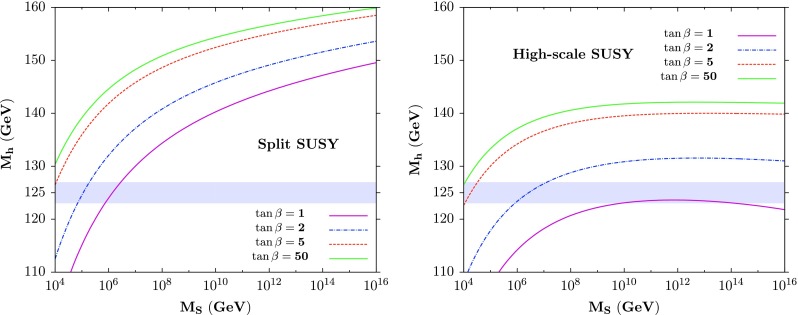
The value of $h$ boson mass as a function of the SUSY scale $M_S$ for several values of $\tan \beta =1,2,5,50$ in the split-SUSY (*left*) and high-scale SUSY (*right*) scenarios. From Ref. [35]

### Split and high-scale SUSY models

In the preceding discussion, we have always assumed that the SUSY-breaking scale is relatively low, $M_S \lesssim 3$ TeV, which implies a natural SUSY scenario [33] with supersymmetric and heavier Higgs particles that could be observed at the LHC. However, as already mentioned, this choice is mainly dictated by fine-tuning considerations which are a rather subjective matter as there is no compelling criterion to quantify the acceptable amount of tuning. One could well abandon the SUSY solution to the hierarchy problem and have a very high $M_S$, which implies that, except for the lightest $h$ boson, no other scalar particle is accessible at the LHC or at any foreseen collider.

This argument has been advocated to construct the so-called split SUSY scenario [76–78] in which the soft SUSY-breaking mass terms for all the scalars of the theory, except for one Higgs doublet, are extremely large, i.e. their common value $M_S$ is such that $M_S \gg 1$ TeV (such a situation occurs e.g. in some string motivated models [96]). Instead, the mass parameters for the spin-$\frac{1}{2}$ particles, the gauginos and the higgsinos, are left in the vicinity of the EWSB scale, allowing for a solution to the dark matter problem and a successful gauge coupling unification, the two other SUSY virtues. The split SUSY models are much more predictive than the usual pMSSM as only a handful parameters are needed to describe the low-energy theory. Besides the common value $M_S$ of the soft SUSY-breaking sfermion and one Higgs mass parameters, the basic inputs are essentially the three gaugino masses $M_{1,2,3}$ (which can be unified to a common value at $M_\mathrm{GUT}$ as in mSUGRA), the higgsino parameter $\mu$ and $\tan \beta$. The trilinear couplings $A_f$, which are expected to have values close to the EWSB scale set by the gaugino/higgsino masses that are much smaller than $M_S$, will play a negligible role.

Concerning the Higgs sector, the main feature of split SUSY is that at the high scale $M_S$, the boundary condition on the quartic Higgs coupling is determined by SUSY:

8 $$\begin{aligned} \lambda (M_S) = \frac{1}{4}\left[ g^2(M_S)+g^{\prime 2}(M_S) \right] \,\cos ^22\beta . \end{aligned}$$

where $g$ and $g'$ are the SU(2) and U(1) gauge couplings. Here, $\tan \beta$ is not a parameter of the low-energy effective theory as it enters only the boundary condition above and cannot be interpreted as the ratio of the two Higgs vevs.

If the scalars are very heavy, they will lead to radiative corrections in the Higgs sector that are significantly enhanced by large logarithms, $\log ( M_S/M_\mathrm{EWSB})$ where $M_\mathrm{EWSB} \approx |\mu |, M_{2}$. In order to have reliable predictions, one has to properly decouple the heavy states from the low-energy theory and resum the large logarithmic corrections; in addition, the radiative corrections due to the gauginos and the higgsinos have to be implemented. Following the early work of Ref. [76–78], a comprehensive study of the split SUSY spectrum has been performed in Ref. [97]. All the features of the model have been implemented in the code SuSpect [82] upon which the analysis presented in Ref. [35] and summarised here is based.

One can adopt an even more radical attitude than in split SUSY and assume that the gauginos and higgsinos are also very heavy, with a mass close to the scale $M_S$; this is the case in the so-called high-scale SUSY model [79, 80]. Here, one abandons not only the SUSY solution to the fine-tuning problem but also the solution to the dark matter problem by means of the LSP and the successful unification of the gauge couplings. However, there will still be a trace of SUSY at low energy: the matching of the SUSY and low-energy theories is indeed encoded in the Higgs quartic coupling $\lambda$ of Eq. (8). Hence, even if broken at very high scales, SUSY would still lead to a “light” Higgs whose mass will give information on $M_S$ and $\tan \beta$.

The treatment of the Higgs sector of the high-scale SUSY scenario is similar to that of split SUSY: one simply needs to decouple the gauginos and higgsinos from the low-energy spectrum (in particular remove their contributions to the renormalisation group evolution of the gauge and Yukawa couplings and to the radiative corrections to $M_h$) and set their masses to $M_S$. The version of the program Suspect which handles the split SUSY case can be adapted to also cover the $M_1 \approx M_2 \approx M_3 \approx |\mu | \approx M_S$ case.

Using this tool, a scan in the $[\tan \beta , M_S]$ plane has been performed to determine the value of $M_h$ in the split SUSY and high-scale SUSY scenarios; in the former case, $M_\mathrm{EWSB} \approx \sqrt{|M_2\mu | } \approx 246$ GeV was chosen for the low scale. The results are shown in Fig. 3, where $M_h$ is displayed as a function of $M_S$ for selected values of $\tan \beta$ in both split (left plot) and high-scale (right plot) SUSY.

As expected, the maximal $M_h$ values are obtained at high $\tan \beta$ and $M_S$ values and, at the scale $M_S \approx 10^{16}$ GeV at which the couplings $g$ and $g'$ approximately unify in the split SUSY scenario, one obtains $M_h \approx 160$ GeV for the higher $\tan \beta =50$ value. Not included is the error bands in the SM inputs that would lead to an uncertainty of about 2 GeV on $M_h$, which is now mainly due to the 1 GeV uncertainty on $m_t$. In addition, the zero-mixing scenario was assumed as the parameter $A_t$ is expected to be much smaller than $M_S$; this approximation might not be valid for $M_S$ values below 10 TeV and a maximal mixing $A_t/M_S = \sqrt{6}$ would increase the Higgs mass value by up to 10 GeV at $M_S ={\mathcal {O}} (1~\mathrm{TeV})$ as was discussed earlier for the pMSSM. In the high-scale SUSY scenario, one obtains a value $M_h\approx 142$ GeV (with again an uncertainty of approximately 2 GeV from the top mass) for high $\tan \beta$ values and at the unification scale $M_S \approx 10^{14}$ GeV [79, 80]. Much smaller $M_h$ values, in the 120 GeV range, can be obtained for lower scales and $\tan \beta$.

Hence, the requirement that the Higgs mass is in the range 123 $\lesssim M_h \lesssim 127$ GeV imposes strong constraints on the parameters of these two models. For this mass range, very large scales are needed for $\tan \beta \approx 1$ in the high-scale SUSY scenario, while scales not too far from $M_S \approx 10^{4}~\mathrm{GeV}$ are required at $\tan \beta \gg 1$ in both the split and the high-scale scenarios. In this case, SUSY should manifest itself at scales much below $M_\mathrm{GUT}$ if $M_h\approx 125$ GeV.

### Splitting the Higgs and sfermion sectors

In the previous high-scale scenarios, the Higgs mass parameters were assumed to be related to the mass scale of the scalar fermions in such a way that the masses of the heavier Higgs particles are also of the order of the SUSY scale, $M_A \approx M_S$. However, this needs not to be true in general and one can, for instance, have a NUHM-like scenario where the Higgs masses are decoupled from those of the sfermions. If one is primarily concerned with the MSSM Higgs sector, one may be rather conservative and allow any value for $M_A$ irrespective of the SUSY-breaking scale $M_S$. This is the quite “model-independent” approach that has been advocated in Refs. [98–101]: take $M_A$ as a free parameter of the pMSSM, with values ranging from ${\mathcal {O}}( 100$ GeV) up to ${\mathcal {O}}(M_S)$, but make no restriction on $M_S$, which can be set to any value, even very high.

**Fig. 4:**
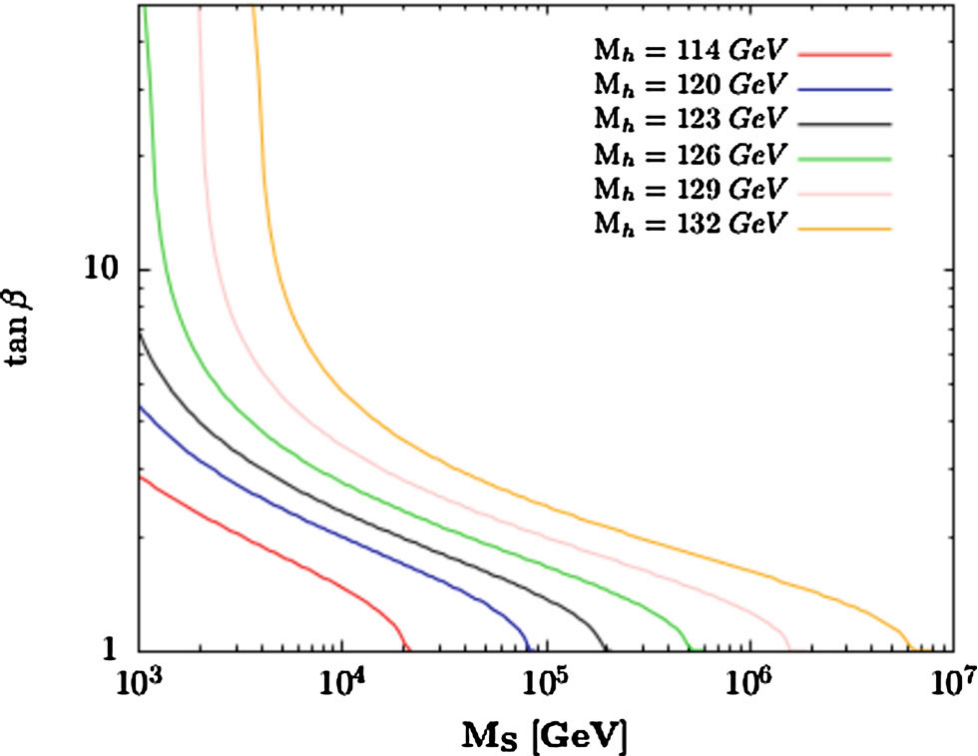
Contours for fixed values $M_h=120, 123, 126,129$ and 132 GeV in the $[\tan \beta , M_S]$ plane in the decoupling limit $M_A \gg M_Z$; the “LEP2 contour” for $M_h=114$ GeV is also shown

An important consequence of this possibility is that it reopens the low $\tan \beta$ region, $\tan \beta \lesssim 3$, which was long thought to be forbidden if one requires a SUSY scale $M_S \lesssim 1$ TeV, as a result of the limit $M_h \gtrsim 114$ GeV from the negative search of a SM-like Higgs boson at LEP [31]. If the SUSY scale is large enough, these small $\tan \beta$ values would become viable again. To estimate the required magnitude of $M_S$, one can still use Suspect in which the possibility $M_S \gg 1$ TeV is implemented [97] with the full set of radiative corrections up to two loops included. In Fig. 4, displayed are the contours in the plane $[\tan \beta , M_S]$ for fixed mass values $M_h=120$–132 GeV of the observed Higgs state (these include a 3 GeV theoretical uncertainty and also a 3 GeV uncertainty on the top quark mass [84] that is conservatively added linearly in the extreme cases). The maximal mixing $X_t = \sqrt{6} M_S$ scenario is assumed with 1 TeV gaugino/higgsino mass parameters.

One observes that values of $\tan \beta \approx 1$ are possible and allow for an acceptable $M_h$ value provided the scale $M_S$ is large enough. For instance, while one can accommodate a scale $M_S \approx 1$ TeV with $\tan \beta \approx 5$, a large scale $M_S \approx 20$ TeV is required to obtain $\tan \beta \approx 2$; to reach the limit $\tan \beta =1$, an order of magnitude increase of $M_S$ will be required. Outside the decoupling regime, the obtained $M_S$ for a given $M_h$ value will be of course larger. For completeness, also shown is the contour for the LEP2 limit $M_h=114$ GeV which illustrates the fact that $\tan \beta \approx 1$ is still allowed provided that $M_S \gtrsim 20$ TeV.

### A new parametrisation of the Higgs sector

It was pointed out in Refs. [98, 102–104] that when the measured value of the $h$ boson mass $M_h=125$ GeV is taken into account, the MSSM Higgs sector with solely the dominant radiative corrections included can be again described with only two free parameters such as $\tan \beta$ and $M_A$ as was the case at tree level. In other words, the dominant radiative corrections that involve the SUSY parameters are fixed by the value of $M_h$. This observation leads to a rather simple parametrisation of the MSSM Higgs sector.

More specifically, let us assume that in the $2\times 2$ matrix for the radiative corrections to the CP-even Higgs mass matrix Eq. (1), only the leading $\varDelta {\mathcal {M}}^{2}_{22}$ entry of Eq. (5) that involves the by far dominant stop–top sector contribution is taken into account; this is the so-called $\epsilon$ approximation and its refinements [15, 16, 28]. In this $\varDelta {\mathcal {M}}^{2}_{22} \gg \varDelta {\mathcal {M}}^{2}_{11}, \varDelta {\mathcal {M}}^{2}_{ 12}$ limit, one can simply trade $\varDelta {\mathcal {M}}^{2}_{22}$ for the by now known $h$ mass value $M_h=125$ GeV and obtain

9 $$\begin{aligned} \begin{aligned} M_{H}^2&= \frac{\left( M_{A}^2+M_{Z}^2-M_{h}^2\right) \left( M_{Z}^2 c^{2}_{\beta }+M_{A}^2 s^{2}_{\beta }\right) - M_{A}^2 M_{Z}^2 c^{2}_{2\beta } }{M_{Z}^2 c^{2}_{\beta }+M_{A}^2 s^{2}_{\beta } - M_{h}^2} \\ \alpha&= -\arctan \left( \frac{ (M_{Z}^2+M_{A}^2) c_{\beta } s_{\beta }}{M_{Z}^2 c^{2}_{\beta }+M_{A}^2 s^{2}_{\beta } - M_{h}^2}\right) \end{aligned} \end{aligned}$$

This was called the habemus MSSM or hMSSM in Ref. [81].

**Fig. 5:**
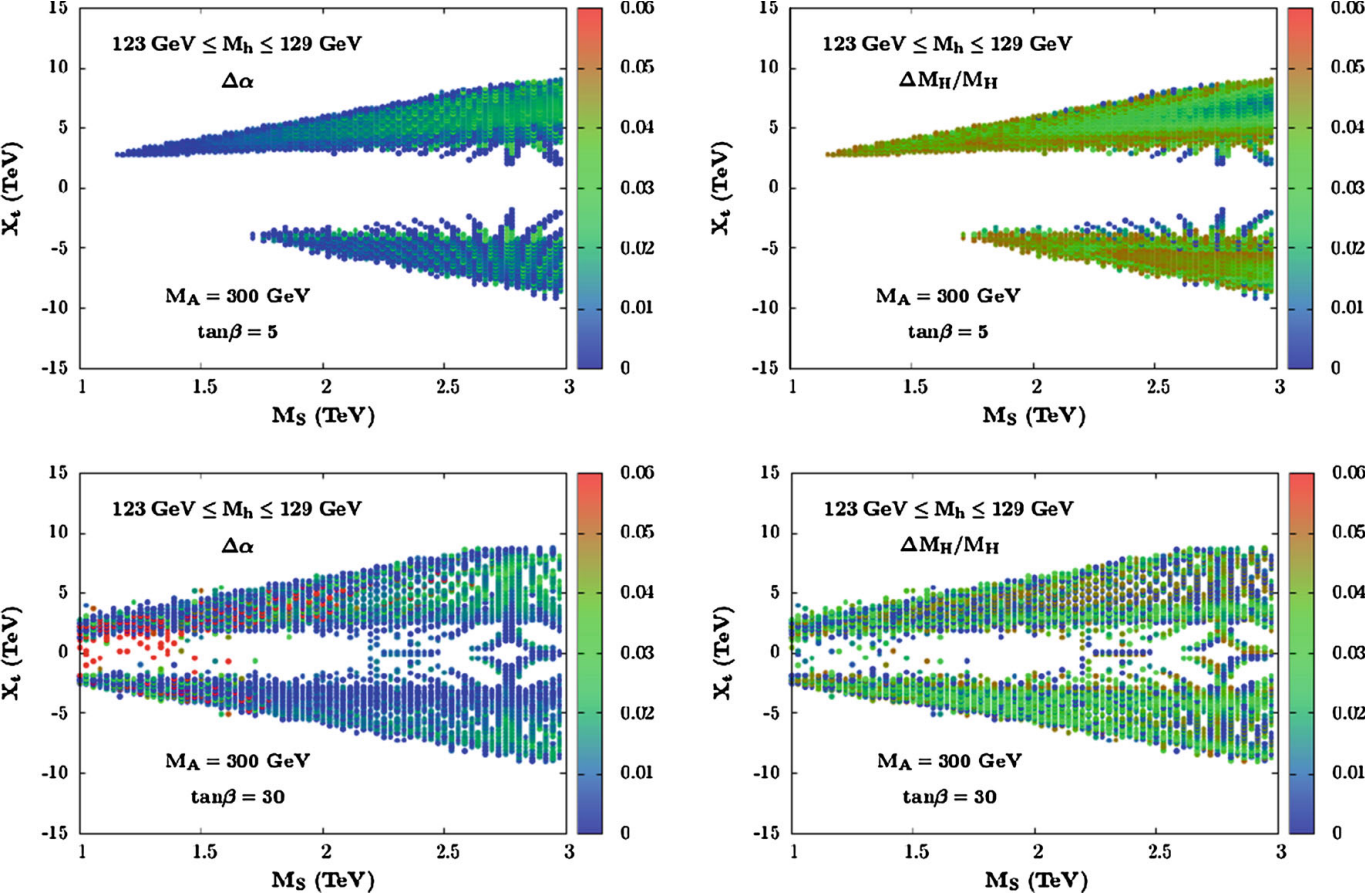
The variation of the mass $M_H$ and the mixing angle $\alpha$ are shown as separate *vertical colored* scales, in the plane $[M_{S},X_{t}]$ when the full two loop corrections are included with and without the subleading matrix elements $\varDelta {\mathcal {M}}^{2}_{11}$ and $\varDelta {\mathcal {M}}^{2}_{12}$. $M_A=300$ GeV, $\tan \beta =5$ or and 30 are taken and the other parameters are varied as described in the text [81]

However, this interesting and simplifying feature has to been demonstrated for all MSSM parameters and, in particular, one needs to prove that the impact of the subleading corrections $\varDelta {\mathcal {M}}^{2}_{11}$ and $\varDelta {\mathcal {M}}^{2}_{12}$ is small. To do so, a scan of the pMSSM parameter space using the program SuSpect, in which the full two-loop radiative corrections to the Higgs sector are implemented, has been performed [81]. For a chosen ($\tan \beta$,$M_A$) input set, the soft-SUSY parameters that play an important role in the Higgs sector are varied in the following ranges: $|\mu |\le 3$ TeV, $|A_t,A_b|\le 3 M_S$, $1$ TeV $\le M_3 \le 3$ TeV and $0.5$ TeV $\le M_S \le 3$ TeV ($\approx$3 TeV is the scale up to which programs such as SuSpect are expected to be reliable). The usual GUT relation between the weak scale gaugino masses $6 M_1= 3 M_2 = M_3$ has been assumed and $A_u,A_d, A_\tau = 0$ has been set (these last parameters have little impact on the radiative corrections). The MSSM Higgs sector parameters have been computed all across the parameter space, selecting the points which satisfy the constraint $123 \le M_h \le 129$ GeV when uncertainties are included. For each of theses points, the Higgs parameters have been compared to those obtained in the simplified MSSM approximation, $\varDelta {\mathcal {M}}^{2}_{11} = \varDelta {\mathcal {M}}^{2}_{12} = 0$, with the lightest Higgs boson mass as input. While the requirement that ${M}_h$ should lie in the range 123–129 GeV has been made, $M_h$ was allowed to be different from the one obtained in the “exact” case $\varDelta {\mathcal {M}}^{2}_{11}, \varDelta {\mathcal {M}}^{2}_{12} \ne 0$.

Displayed in Fig. 5 are the differences between the values of the mass $M_H$ and the mixing angle $\alpha$ that are obtained when the two possibilities $\varDelta {\mathcal {M}}^{2}_{11}= \varDelta {\mathcal {M}}^{2}_{12} = 0$ and $\varDelta {\mathcal {M}}^{2}_{11}, \varDelta {\mathcal {M}}^{2}_{12} \ne 0$ are considered. This is shown in the plane $[M_{S},X_{t}]$ with $X_t=A_t-\mu \cot \beta$ when all other parameters are scanned as above. The $A$ boson mass was fixed to $M_A=300$ GeV (a similar result was obtained for $M_A\approx 1$ TeV) and two representative values $\tan \beta =5$ and $30$ are used. The conservative approach of plotting only points which maximise these differences has been adopted.

In all cases, the difference between the two $M_H$ values is very small (in fact, much smaller than the $H$ boson total decay width $\varGamma _H$), less than a few percent, while for $\alpha$ the difference does not exceed $\approx$0.025 for low values of $\tan \beta$, but at high $\tan \beta$ values, one can reach the level of $\approx$0.05 in some rare situations (large values of $\mu$, which enhance the $\mu \tan \beta$ contributions). Nevertheless, at high enough $\tan \beta$, we are far in the decoupling regime already for $M_A \gtrsim 200$ GeV and such a difference does not significantly affect the couplings of the $h$ and $H$ bosons which, phenomenologically, are the main ingredients.

Hence, even when including the full set of radiative corrections, it remains a good approximation to use Eq. (9) to derive the parameters $M_H$ and $\alpha$ in terms of the inputs $\tan \beta , M_A$ and the measured $M_h$ value.

**Fig. 6:**
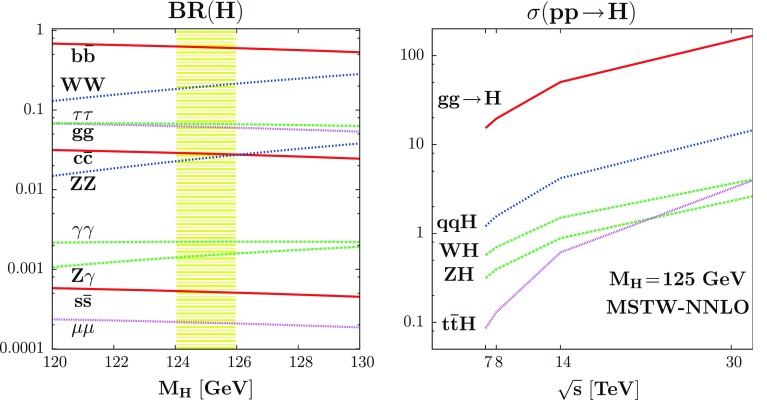
The SM-like Higgs boson branching ratios in the mass range $120$–130 GeV (*left*) and its production cross sections at proton colliders as a function of the c.m. energy (*right*) [107]

In the case of the charged Higgs boson (whose physics is described by $\tan \beta , M_{H^\pm }$ and eventually $\alpha$), the radiative corrections to $M_{H^\pm }$ are much smaller for large enough $M_A$ and one has, at the few percent level in most cases (which is again smaller than the total $H^\pm$ decay width),

10 $$\begin{aligned} M_{H^\pm } \simeq \sqrt{ M_A^2 + M_W^2}. \end{aligned}$$

In conclusion, this approximation allows to ignore the radiative corrections to the Higgs masses and their complicated dependence on the MSSM parameters and to use a simple formula to derive the other parameters of the Higgs sector, $\alpha$, $M_H$ as well as $M_{H^\pm }$. This considerably simplifies phenomenological analyses in the MSSM Higgs sector which up to now rely either on large scans of the parameter space (as in the previous subsections) or resort to benchmark scenarios in which most of the MSSM parameters are fixed (as is the case of Ref. [105] for instance).

## Implications of the Higgs production rates

### Light Higgs decay and production at the LHC

In many respects, the Higgs particle was born under a very lucky star as the mass value of $\approx$125 GeV (although too high for a natural SUSY) allows to produce it at the LHC in many redundant channels and to detect it in a variety of decay modes. This allows detailed studies of the Higgs properties as will be discussed in this section.

We start by summarizing the production and decay at the LHC of a light SM-like Higgs particle, which should correspond to the lightest MSSM $h$ boson in the decoupling regime. First, for $M_h \approx 125$ GeV, the Higgs mainly decays into $b \bar{b}$ pairs but the decays into $WW^*$ and $ZZ^*$ final states, before allowing the gauge bosons to decay leptonically $W \rightarrow \ell \nu$ and $Z \rightarrow \ell \ell$ ($\ell = e,\mu$), are also significant. The $h \rightarrow \tau ^+\tau ^-$ channel (as well as the $gg$ and $c\bar{c}$ decays that are not detectable at the LHC) is also of significance, while the clean loop induced $h\rightarrow \gamma \gamma$ mode can be easily detected albeit its small rates. The very rare $h\rightarrow Z\gamma$ and even $h\rightarrow \mu ^+\mu ^-$ channels should be accessible at the LHC but only with a much larger data sample. This is illustrated in the left-hand side of Fig. 6, where the decay branching fractions of a SM-like Higgs are displayed for the narrow mass range $M_h=120$–130 GeV

On the other hand, many Higgs production processes have significant cross sections as is shown in the right-hand side of Fig. 6, where they are displayed at a proton collider at various past, present and foreseen center of mass energies for a 125 GeV SM-like Higgs boson; the MSTW parton densities [106] have been used.

**Fig. 7:**
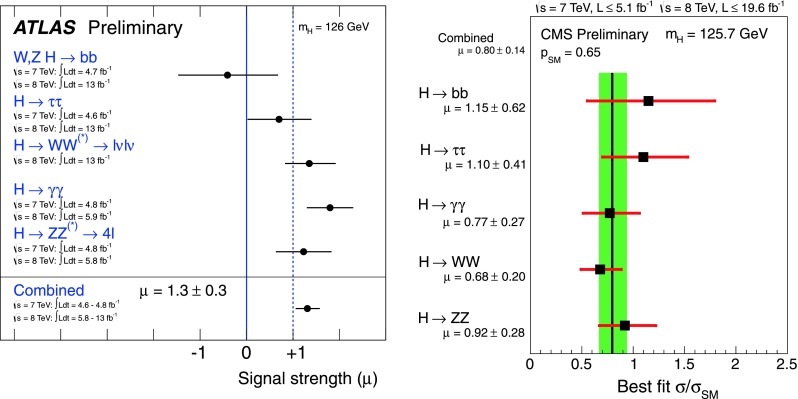
The signal strengths on the SM Higgs boson in the various search channels provided by ATLAS [108] and CMS [109] with the data collected so far at $\sqrt{s}=7$+8 TeV

While the by far dominant gluon fusion mechanism $gg\rightarrow h$ (ggF) has extremely large rates ($\approx$20 pb at $\sqrt{s}= 7$–8 TeV), the subleading channels, i.e. the vector boson fusion (VBF) $qq \rightarrow hqq$ and the nHiggs-strahlung (HV) $q\bar{q} \rightarrow hV$ with $V=W,Z$ mechanisms, have cross sections which should allow for a study of the Higgs particle already at $\sqrt{s}\gtrsim 8$ TeV with the amount of integrated luminosity, $\approx$25 fb$^{-1}$, which has been collected by each experiment. The Higgs–top associated process $p p\rightarrow t\bar{t} h$ (ttH) would require higher energy and luminosity.

This pattern already allows the ATLAS and CMS experiments to observe the Higgs boson in several channels and to measure some its couplings in a reasonably accurate way. The channels that have been searched are $h \rightarrow ZZ^* \rightarrow 4\ell ^\pm , h \rightarrow WW^* \rightarrow 2\ell 2 \nu , h \rightarrow \gamma \gamma$ where the Higgs is mainly produced in ggF with subleading contributions from $hjj$ in the VBF process, $h \rightarrow \tau \tau$ where the Higgs is produced in association with one (in ggF) and two (in VBF) jets, and finally $h\rightarrow b \bar{b}$ with the Higgs produced in the HV process. One can ignore for the moment the additional search channels $h \rightarrow \mu \mu$ and $h \rightarrow Z\gamma$ for which the sensitivity is still too low with the data collected so far.

A convenient way to scrutinise the couplings of the produced $h$ boson is to consider their deviation from the SM expectation. One then considers for a given search channel the signal strength modifier $\mu$, which, with some approximation, can be identified with the Higgs production cross section times decay branching fractions normalised to the SM value. For the $h \rightarrow XX$ decay channel, one would have in the narrow width approximation,

11 $$\begin{aligned} \mu _{XX}\vert _\mathrm{th}&= \frac{\sigma ( pp \rightarrow h \rightarrow XX)}{ \sigma ( pp \rightarrow h \rightarrow XX)|_\mathrm{SM}} \nonumber \\&= \frac{\sigma ( pp \rightarrow h)\times \mathrm{BR} (h \rightarrow XX)}{\sigma ( pp \rightarrow h)|_\mathrm{SM} \times \mathrm{BR} (h \rightarrow XX)|_\mathrm{SM} }. \end{aligned}$$

which, from the experimental side would correspond to

12 $$\begin{aligned} \mu _{XX}\vert _\mathrm{exp} \simeq \frac{N^\mathrm{ev}_{XX}}{ \epsilon \times \sigma ( pp \rightarrow h)|_\mathrm{SM} \times \mathrm{BR} (h \rightarrow XX)|_\mathrm{SM} \times {\mathcal {L}}}\nonumber \\ \end{aligned}$$

where $N^\mathrm{ev}_{XX}$ is the measured number of events in the $XX$ channel, $\epsilon$ the selection efficiency and ${\mathcal {L}}$ the luminosity.

ATLAS and CMS have provided the signal strengths for the various final states with a luminosity of, respectively, $\approx$5 fb$^{-1}$ for the 2011 run at $\sqrt{s}=7$ TeV and $\approx$20 fb$^{-1}$ for the 2012 run at $\sqrt{s}=8$ TeV. The constraints given by the two collaborations are shown in Fig. 7.

When the various analysed Higgs search channels are combined, this leads to a global signal strength [108, 109]

13 $$\begin{aligned} \mathrm{ATLAS}:&\mu _\mathrm{tot} =1.30 \pm 0.30\, \nonumber \\ \mathrm{CMS}:&\mu _\mathrm{tot} =0.87 \pm 0.23 \, \end{aligned}$$

which shows a good agreement with the SM expectation. In fact, when the ATLAS and CMS values are combined, one finds a global signal strength that is very close to unity, implying that the observed Higgs is rather SM-like.

Hence, already with the rather limited statistics at hand, the accuracy of the measurements in Eq. (13) is reaching the 20 % level for the ATLAS and CMS collaborations. This is at the same time impressive and worrisome. Indeed, as mentioned earlier the main Higgs production channel is the top and bottom quark loop mediated gluon fusion mechanism and, at $\sqrt{s}=7$ or 8 TeV, the three other mechanisms contribute at a level below 15 % when their rates are added and before kinematical cuts are applied. The majority of the signal events presently observed at the LHC, in particular in the main search channels $h \rightarrow \gamma \gamma , h \rightarrow ZZ^* \rightarrow 4\ell , h \rightarrow WW^* \rightarrow 2 \ell 2\nu$ and, to a lesser extent, $h \rightarrow \tau \tau$, thus come from the ggF mechanism which is known to be affected by large theoretical uncertainties.

As a matter of fact, although the cross section $$\sigma (gg\rightarrow h)$$ is known up next-to-next-to-leading order (NNLO) in perturbative QCD (and at least at NLO for the electroweak interaction) [110], there is a significant residual scale dependence which points to the possibility that still higher order contributions beyond NNLO cannot be totally excluded. In addition, as the process is of $${\mathcal {O}}(\alpha _s^2)$$ at LO and is initiated by gluons, there are sizable uncertainties due to the gluon parton distribution function (PDF) and the value of the coupling $$\alpha _s$$. A third source of theoretical uncertainties, the use of an effective field theory (EFT) approach to calculate the radiative corrections beyond the NLO approximation, should in principle also be considered [111, 112]. In addition, large uncertainties arise when the $$gg \rightarrow h$$ cross section is broken into the jet categories $$h + 0j, h + 1j$$ and $$ h + 2j$$ [113]. In total, the combined theoretical uncertainty has been estimated to be of order $$\varDelta ^{ \mathrm th} \approx \pm 15\,\%$$ by the LHC Higgs cross section working group [110] and it would increase up to $$\varDelta ^\mathrm{th} \approx \pm $$20 % if the EFT uncertainty is also included^2^ [112].

Hence, the theoretical uncertainty is already at the level of the accuracy of the cross section measured by the ATLAS and CMS collaborations, Eq. (13). Another drawback of the analyses is that they involve strong theoretical assumptions on the total Higgs width since some contributing decay channels not accessible at the LHC are assumed to be SM-like and possible invisible Higgs decays in scenarios beyond the SM are supposed not to occur.

In Ref. [107], following earlier work [114–117], it has been suggested to consider the decay ratios $D_{XX}$ defined as

14 $$\begin{aligned} D_{XX}^\mathrm{p}&= \frac{\sigma ^\mathrm{p} ( pp \rightarrow h \rightarrow XX)}{ \sigma ^\mathrm{p} ( pp \rightarrow h \rightarrow VV)} \end{aligned}$$

15 $$\begin{aligned}&= \frac{\sigma ^\mathrm{p} ( pp \rightarrow h)\times \mathrm{BR} (h \rightarrow XX)}{ \sigma ^\mathrm{p}( pp \rightarrow h) \times \mathrm{BR} (h \rightarrow VV)}\end{aligned}$$

16 $$\begin{aligned}&= \frac{\varGamma ( h \rightarrow XX)}{ \varGamma ( h \rightarrow VV)} \propto \frac{c_{X}^2}{c_{V}^2} \end{aligned}$$

for a specific production process $\mathrm{p= ggF, VBF, VH}$ or all (for inclusive production) and for a given decay channel $h\rightarrow XX$ when the reference channel $h\rightarrow VV$ is used. In these ratios, the cross sections $\sigma ^p (pp \rightarrow h)$ and hence, their significant theoretical uncertainties will cancel out, leaving out only the ratio of decay branching fractions and hence of partial decay widths. These can be obtained with the program HDECAY [118] which includes all higher order effects and are affected by much smaller uncertainties. Thus, the total decay width which includes contributions from channels not under control such as possible invisible Higgs decays, do not appear in the ratios $D_{XX}^\mathrm{p}$. Some common experimental systematical uncertainties such as the one from the luminosity measurement and the small uncertainties in the Higgs decay branching ratios also cancel out. We are thus, in principle, left with only with the statistical uncertainty and some (non common) systematical errors. The ratios $D_{XX}$ involve, up to kinematical factors and known radiative corrections, only the ratios $\vert c_X \vert ^2/$ $\vert c_V\vert ^2$ of the Higgs reduced couplings to the particles $X$ and $V$ compared to the SM expectation, $c_X \equiv g_{hXX}/g_{hXX}^\mathrm{SM}$.

For the time being, three independent ratios can be considered: $D_{\gamma \gamma }, D_{\tau \tau }$ and $D_{bb}$. $D_{\gamma \gamma }$ is the ratio of the inclusive ATLAS and CMS di-photon and $ZZ$ channels that are largely dominated by the ggF mechanism; $D_{\tau \tau }$ is the signal strength ratio in the $\tau \tau$ and $WW$ searches where one selects Higgs production in ggF with an associated jet or in the VBF production mechanism; $D_{bb}$ is the ratio of the $h\rightarrow b\bar{b}$ and $h\rightarrow WW$ decays in $hV$ production for which the sensitivities are currently too low.

In order to test the compatibility of the couplings of the $M_h=125$ GeV Higgs state with the SM expectation, one can perform a fit based on the $\chi _R^2$ function

17 $$\begin{aligned} \chi ^2_R&= \frac{\left[ D_{\gamma \gamma }^{ggF}-\frac{\mu _{\gamma \gamma }}{\mu _{ZZ}} \vert ^{ggF}_\mathrm{exp}\right] ^2}{\left[ \delta \left( \frac{\mu _{\gamma \gamma }}{\mu _{ZZ}}\right) _{ggF}\right] ^2} +\frac{\left[ D_{bb}^{VH}-\frac{\mu _{bb}}{\mu _{WW}}\vert ^{Vh}_\mathrm{exp}\right] ^2}{\left[ \delta \left( \frac{\mu _{bb}}{\mu _{WW}}\right) _{Vh}\right] ^2} \nonumber \\&+\frac{\left[ D_{\tau \tau }^{ggF}-\frac{\mu _{\tau \tau }}{\mu _{WW}}\vert ^{ggF}_\mathrm{exp}\right] ^2}{\left[ \delta \left( \frac{\mu _{\tau \tau }}{\mu _{WW}}\right) _{ggF} \right] ^2}\! +\! \frac{\left[ D_{\tau \tau }^\mathrm{VBF}\!-\!\frac{\mu _{\tau \tau }}{\mu _{WW}}\vert ^\mathrm{VBF}_\mathrm{exp}\right] ^2}{\left[ \delta \left( \frac{\mu _{\tau \tau }}{\mu _{WW}}\right) _\mathrm{VBF}\right] ^2}\nonumber \\ \end{aligned}$$

The errors $\delta (\mu _{XX}/{\mu _{VV}})$ are computed assuming no correlations between the different final state searches. The uncertainties on the ratios are derived from the individual errors that are dominated by the experimental uncertainties as one expects that the theoretical uncertainties largely cancel out in the ratios $D_{\gamma \gamma }$, $D_{bb}$ and $D_{\tau \tau }$.

For the signal strengths above, the theoretical uncertainties have to be treated as a bias (and not as if they were associated with a statistical distribution) and the fit has to be performed for the two extremal values of the signal strengths: $\mu _{i} \vert _{ \mathrm exp} \pm \delta \mu _i/\mu _i \vert _\mathrm{th}$ with the theoretical uncertainty $\delta \mu _i/ \mu _i \vert _\mathrm{th}$ conservatively assumed to be $\pm 20\,\%$ for both the gluon and the vector boson fusion mechanisms (because of the contamination due to $gg\rightarrow h+2j$ in the latter case) and $\approx$5 % for $hV$ associated production.

### Fit of the Higgs couplings and their ratios

A large number of analyses of the Higgs couplings from the LHC data have been performed in the SM and its extensions and a partial list is given in Refs. [119–150].

In the MSSM, the couplings of the CP-even Higgs particles $h$ and $H$ to gauge bosons and fermions, compared to the SM Higgs couplings, are changed by factors that involve the sine and the cosine of the mixing angles $\beta$ and $\alpha$. Outside the decoupling regime where they reach unity, the reduced couplings (i.e. normalised to their SM values) of the lighter $h$ state to third generation $t,b,\tau$ fermions and gauge bosons $V = W/Z$ are for instance given by

18 $$\begin{aligned} c_V^0 = \sin (\beta - \alpha ) , c_t^0 = \cos \alpha / \sin \beta , c_b^0 = - \sin \alpha / \cos \beta \nonumber \\ \end{aligned}$$

They thus depend not only on the two inputs $[\tan \beta , M_A]$, as occurs at tree level, but, a priori, on the entire MSSM spectrum as a result of the radiative corrections, in the same way as the Higgs masses. In principle, as discussed earlier, knowing $\tan \beta$ and $M_A$ and fixing $M_h$ to its measured value, the couplings can be determined in general. However, this is true when only the radiative corrections to the Higgs masses are included. Outside the regime in which the pseudoscalar $A$ boson and the supersymmetric particles are very heavy, there are also direct radiative corrections to the Higgs couplings not contained in the mass matrix of Eq. (1) and which can alter this simple picture.

First, in the case of $b$-quarks, additional one-loop vertex corrections modify the tree–level $h b \bar{b}$ coupling: they grow as $m_b \mu \tan \beta$ and can be very large at high $\tan \beta$. The dominant component comes from the SUSY–QCD corrections with sbottom–gluino loops that can be approximated by $\varDelta _b \simeq 2\alpha _s/(3\pi ) \times \mu m_{\tilde{g}} \tan \beta /\mathrm{max} (m_{\tilde{g}}^2, m_{\tilde{b}_1}^2,m_{\tilde{b}_2}^2)$ [151]. Outside the decoupling regime the $c_b$ coupling reads

19 $$\begin{aligned} c_b \approx c_b^0 \times [1- \varDelta _b/(1+\varDelta _b) \times (1+ \cot \alpha \cot \beta )] \end{aligned}$$

with $\tan \alpha \rightarrow -1/\tan \beta$ for $M_A \gg M_Z$. A large $\varDelta _b$ would significantly alter the dominant $h \rightarrow b\bar{b}$ partial width and affect the branching fractions of all other decay modes.

In addition, the $ht\bar{t}$ coupling is derived indirectly from the $gg \rightarrow h$ production cross section and the $h \rightarrow \gamma \gamma$ decay branching ratio, two processes that are generated by triangular loops. In the MSSM, these loops involve not only the top quark (and the $W$ boson in the decay $h\rightarrow \gamma \gamma$) but also contributions from supersymmetric particles, if not too heavy. In the case of $gg\rightarrow h$ production, only the contributions of stops is generally important. Including the later and working in the limit $M_h \ll m_t, m_{\tilde{t}_{1,2} }$, the coupling $c_t$ from the ggF process^3^ is approximated by [152]

20 $$\begin{aligned} c_t \approx c_t^0 \bigg [ 1 + \frac{m_t^2}{ 4 m_{\tilde{t}_1}^2 m_{\tilde{t}_2}^2 } \left( m_{\tilde{t}_1}^2 + m_{\tilde{t}_2}^2 - X_t^2\right) \bigg ] \end{aligned}$$

which shows that indeed, $\tilde{t}$ contributions can be very large for light stops and for large stop mixing. In the $h \rightarrow \gamma \gamma$ decay rate, because the $t, \tilde{t}$ electric charges are the same, the $ht\bar{t}$ coupling is shifted by the same amount. If one ignores the usually small contributions of the other sparticles (to be discussed in the next subsection), the $ht\bar{t}$ vertex can be simply parametrised by the effective coupling of Eq. (20).

We note that the $h$ couplings to $\tau$ leptons and $c$ quarks do not receive the direct corrections of Eqs. (19) and (20) and one should still have $c_c=c_t^0$ and $c_\tau = c_b^0$. However, using $c_{t,b}$ or $c_{t,b}^0$ in this case has almost no impact in practice as these couplings appear only in the branching ratios for the decays $h \rightarrow c\bar{c}$ and $\tau ^+ \tau ^-$, which are small and the direct corrections should not be too large. One can thus, in a first approximation, assume that $c_c=c_t$ and $c_\tau =c_b$. Another caveat is due to the invisible Higgs decays which are assumed to be absent and which will be discussed later.

Hence, because of the direct corrections, the Higgs couplings cannot be described only by $\beta$ and $\alpha$ as in Eq. (18). To characterise the Higgs particle at the LHC, it was advocated that at least three independent $h$ couplings should be considered, namely $c_t$, $c_b$ and $c_V=c_V^0$ [81]. One can thus define the following effective Lagrangian:

21 $$\begin{aligned} {\mathcal {L}}_h&= c_V g_{hWW} h W_{\mu }^+ W^{- \mu } + c_V g_{hZZ} h Z_{\mu }^0 Z^{0 \mu } - c_t y_t h \bar{t}_L t_R \nonumber \\&- c_t y_c h \bar{c}_L c_R - c_b y_b h \bar{b}_L b_R - c_b y_\tau h \bar{\tau }_L \tau _R + \mathrm{h.c.}\nonumber \\ \end{aligned}$$

where $y_{t,c,b,\tau }=m_{t,c,b,\tau }/v$ are the Yukawa couplings of the heavy SM fermions, $g_{hWW} = 2M^2_W/v$ and $g_{hZZ} = M^2_Z/v$ the $hWW$ and $HZZ$ couplings and $v$ the SM Higgs vev.

**Fig. 8:**
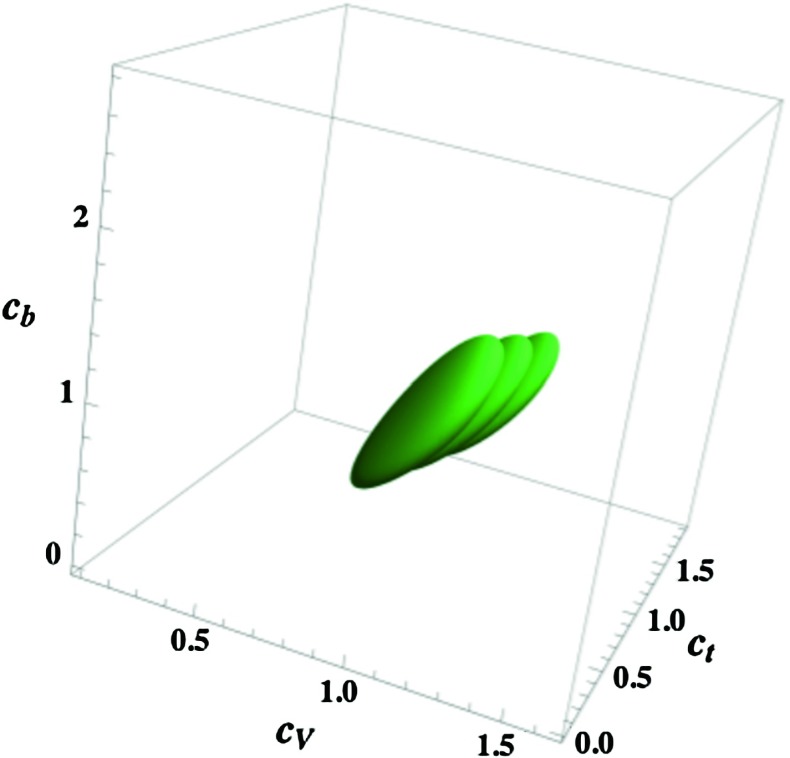
The best-fit region at $68\,\%\,\mathrm{CL}$ for the Higgs signal strengths in the $[c_t,c_b,c_V]$ space [81]. The three overlapping regions are for the central and extreme choices of the theoretical prediction for the Higgs rates including uncertainties

In Ref. [81], a three-dimensional fit of the $h$ couplings was performed in the space $[c_t, c_b, c_V]$, assuming $c_c=c_t$ and $c_\tau =c_b$ as discussed above and of course the custodial symmetry relation $c_V=c_W= c_Z$, which holds in supersymmetric models. The results of this fit are presented in Fig. 8 for $c_t,c_b,c_V \ge 0$. The best-fit value for the couplings, with the $\sqrt{s}=7+$8 TeV ATLAS and CMS data turns out to be $c_t=0.89, c_b=1.01$ and $c_V=1.02$.

**Fig. 9:**
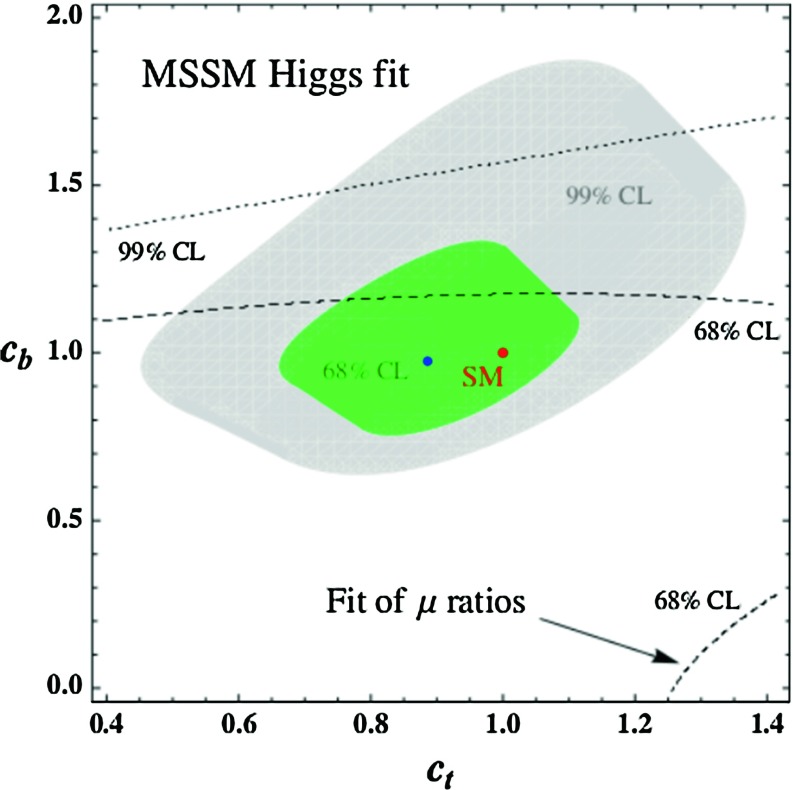
Best-fit regions at $68\,\%$ and $99\,\%\,\mathrm{CL}$ for the Higgs signal strengths and their ratios in the plane $[c_t,c_b]$. The best-fit point is indicated in *blue*. From Ref. [81]

In scenarios where the direct corrections in Eqs. (19) and (20) are not quantitatively significant (i.e. considering either not too large values of $\mu \tan \beta$ or high sfermion masses), one can use the MSSM relations of Eq. (18) to reduce the number of effective parameters down to two. This allows to perform two-parameter fits in the planes $[c_V,c_t]$, $[c_V,c_b]$ and $[c_t,c_b]$. As an example, the fit of the signal strengths and their ratios in the $[c_t,c_b]$ plane is displayed in Fig. 9. In this two-dimensional case, the best-fit point is located at $c_t=0.88$ and $c_b=0.97$, while $c_V \simeq 1$. Note that although for the best-fit point one has $c_b \lesssim 1$, actually $c_b \gtrsim 1$ in most of the $1\sigma$ region.

**Fig. 10:**
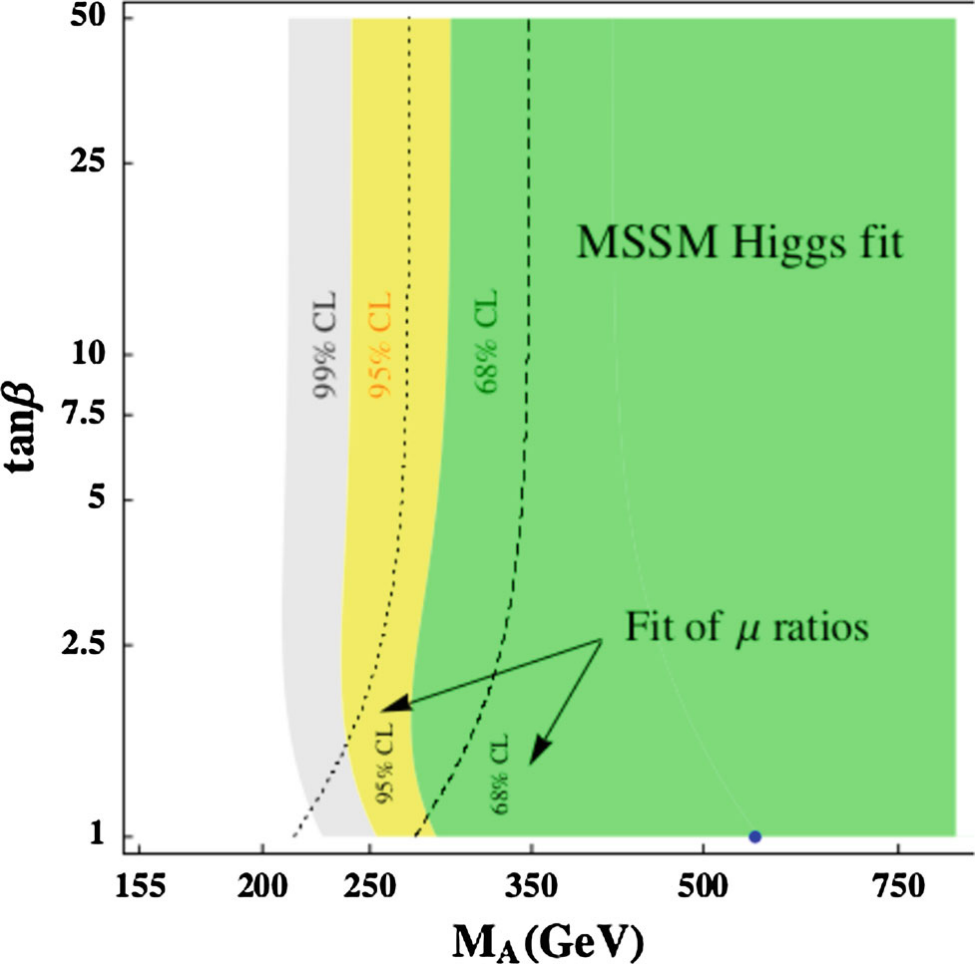
Best-fit regions for the signal strengths and their ratios in the plane $[\tan \beta , M_A]$; the best point is in *blue* [81]

Using the formulae Eq. (9) for the angle $\alpha$ and using the input $M_h \approx 125$ GeV, one can make a fit in the plane $[\tan \beta , M_A]$. This is shown in Fig. 10, where the 68, 95 and 99 % CL contours from the signal strengths and their ratios are displayed when the theory uncertainty is taken as a bias. The best-fit point when the latter uncertainty is set to zero is obtained for the values $\tan \beta = 1$ and $M_A = 557 \; \mathrm{GeV}$, which implies for the other parameters using $M_h=125$ GeV : $M_H= 580$ GeV, $M_{H^\pm }= 563$ GeV and $\alpha =-0.837~\mathrm{rad}$, which leads to $\cos (\beta -\alpha ) \simeq -0.05$. Such a point with $\tan \beta \approx 1$ implies an extremely large value of the SUSY scale, $M_S = {\mathcal {O}}(100)$ TeV, for $M_h\approx 125$ GeV. One should note, however, that the $\chi ^2$ value is relatively stable all over the $1\sigma$ region. Hence, larger values of $\tan \beta$ (and lower values of $M_A$) could also be accommodated reasonably well by the fit, allowing thus for not too large $M_S$ values. In all, cases one has $M_A \gtrsim 200$ GeV though.

### An excess in the $\gamma \gamma$ channel?

In the early LHC data, a significant excess in the $h\rightarrow \gamma \gamma$ detection channel was observed, raising the hope that it could be the first signal for physics beyond the SM. This excess has unfortunately faded away with more statistics and with the full 25 fb$^{-1}$ data collected at $\sqrt{s}=$ 7+8 TeV, there is now only a $\approx 2 \sigma$ excess in ATLAS which measures $\mu _{\gamma \gamma }=1.6\pm 0.3$, while the signal strength measured by the CMS collaboration is $\mu _{\gamma \gamma }= 0.9\pm 0.3$, which is SM-like. Nevertheless, it would be interesting to briefly discuss this excess as, besides the fact that it has triggered a vast literature, the $h \rightarrow \gamma \gamma$ channel is the one where new physics, and SUSY in particular, is most likely to manifest itself.

**Fig. 11:**
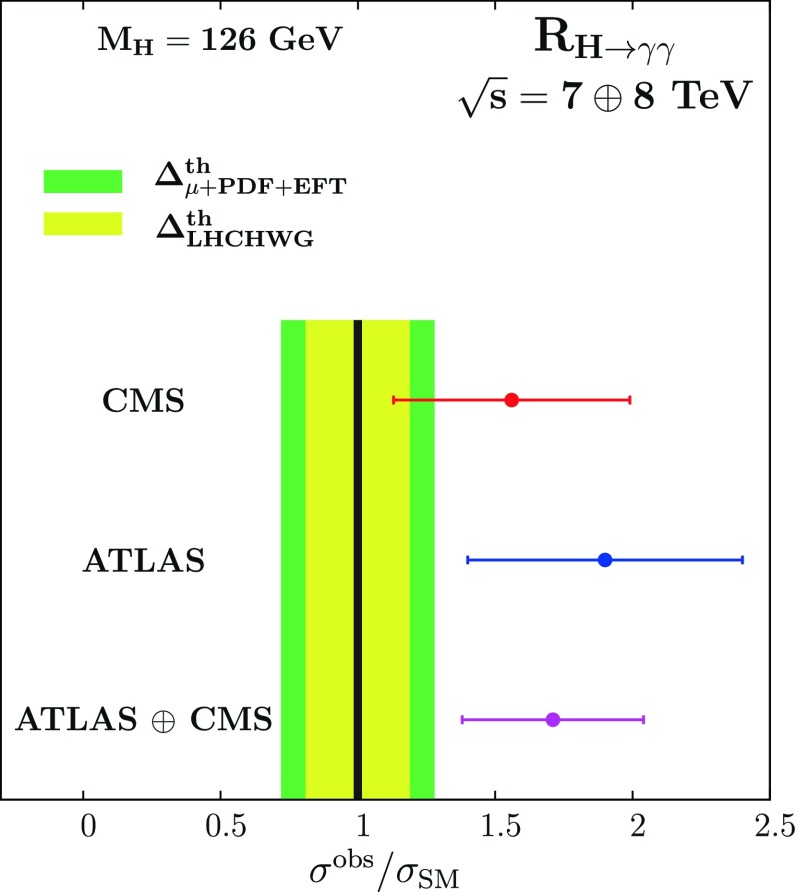
The value of $\mu _{\gamma \gamma }$ given by the ATLAS and CMS collaborations with the $\approx$10 fb$^{-1}$ data collected at $\sqrt{s}=7$ and 8 TeV, as well as their combination, compared to two estimates of the theoretical uncertainty bands; from Ref. [153]

First, it has been realised early that this excess, if not due to a statistical fluctuation, can be easily explained or reduced in the context of the SM by invoking the large theoretical uncertainties that affect the production times decay rate in the dominant channel, $gg\rightarrow h\rightarrow \gamma \gamma$. This is shown in Fig. 11, where the ATLAS and CMS ratios $R_{\gamma \gamma }\equiv \mu _{\gamma \gamma }$ and their combination, obtained with the $\approx 10$ fb$^{-1}$ data collected at $\sqrt{s}=7+$8 TeV, is compared to the theory uncertainty bands obtained by the LHC Higgs group [110] and in Ref. [112]. It is clear that including the theory uncertainty as a bias helps to reduce the discrepancy between measurement and expectation and e.g. the excess reduces to $1.3\sigma$ from the original $\gtrsim 2\sigma$ value.

**Fig. 12:**
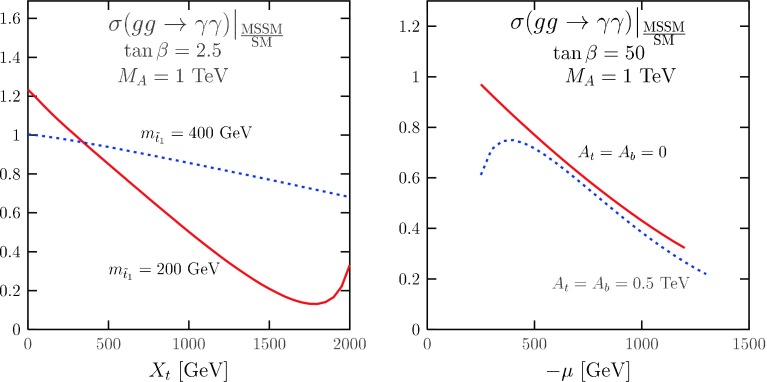
The deviation of $\mu _{\gamma \gamma }$ from its SM expectation from stop (*left*) and sbottom (*right*) contributions in various scenarios to the $\sigma (gg\rightarrow h) \times \mathrm{BR}( h \rightarrow \gamma \gamma )$ rate; from Ref. [152]

Ignoring this option, let us summarise the various possibilities that could explain this excess in the context of the MSSM. Deviations of $\mu _{\gamma \gamma }$ from the SM value may be due to modifications of either the production cross section or the decay branching fraction or to both. The $h$ decay branching fractions may be modified by a change of the $h$ total decay width. Since the dominant decay mode is $h \rightarrow b \bar{b}$, a change of the effective $hb\bar{b}$ coupling by the direct vertex corrections of Eq. (19) outside the decoupling regime can change all other Higgs rates including $h\rightarrow \gamma \gamma$. The total width can also be modified by additional decay channels to SUSY particles and the only ones that are allowed by experimental constraints are invisible decays into the LSP that will be discussed later.

Nevertheless, these two possibilities would not only affect the $h\rightarrow \gamma \gamma$ rate but also those of other channels such as $h \rightarrow ZZ$ where no excess has been observed. It is thus more appropriate to look at deviation in the $h\rightarrow \gamma \gamma$ loop induced decay only. In the MSSM, the $h\rightarrow \gamma \gamma$ process receives contributions from scalar top and bottom quarks, staus and charginos as briefly is summarised below.

- Light stops: as already discussed, the $M_h=125$ GeV constraint requires large $M_S = \sqrt{ m_{{\tilde{t}}_1} m_{{\tilde{t}}_2}}$ and/or $X_t$ values. If $m_{{\tilde{t}}_1} \lesssim 500$ GeV, one should have maximal mixing $X_t \approx \sqrt{6} M_S$ and, in this case, the $h \tilde{t}_1 \tilde{t}_1$ coupling of Eq. (20) is large and leads to a sizeable change of the $gg \rightarrow h \rightarrow \gamma \gamma$ rate; cf. Fig. 12 (left). However, an enhancement of the $h\rightarrow \gamma \gamma$ rate is over-compensated by a suppression of $\sigma (gg\rightarrow h)$ that seems not to occur. $\mu _{\gamma \gamma }$ is enhanced only in the no-mixing case, $X_t \approx 0$, which requires very heavy stops which decouple from the amplitude [57–59, 152].
- Light sbottoms: a light $\tilde{b}_R$ state does not conflict with the $M_h$ value as its corrections to the mass are small. For $m_{{\tilde{b}}_1} \lesssim$ 500 GeV, it contributes to the $hgg$ loop but it reduces the $gg\rightarrow h$ production rate; Fig. 12 (right). In turn, it has little impact on the $h\rightarrow \gamma \gamma$ rate because of the largely dominating $W$ loop and the small $\tilde{b}_1$ electric charge. For $m_{{\tilde{b}}_1}\gtrsim 1$ TeV, as indicated by direct LHC searches, $\mu _{\gamma \gamma }$ is unaffected by sbottoms loops [152].
- Light staus: they lead to the largest contributions and have received most of the attention in the literature; see e.g. Ref. [154–156]. For low $m_{\tilde{\tau }_{L/R}}$ values, a few 100 GeV, and large mixing $X_\tau = A_\tau - \mu \tan \beta$, with $\tan \beta \approx 60$ and $|\mu |$=0.5–1 TeV, the lighter stau state has a mass close to the LEP2 bound, $m_{\tilde{\tau }_1} \approx$ 100 GeV and its coupling to the $h$ boson, $g_{h\tilde{\tau } \tilde{\tau }} \propto m_\tau X_\tau$, is huge. The $\tilde{\tau }_1$ contribution can hence significantly increase BR($h\rightarrow \gamma \gamma$), up to 50 % [154–156], but this occurs only for extreme choices of the parameters.
- Light charginos: the $h \chi _i^+ \chi _i^-$ couplings are in general small and are maximal when the $\chi _i^\pm$ states are almost equal higgsino–wino mixtures. For a mass above 100 GeV and maximal couplings to the $h$ boson, the $\chi ^\pm _1$ contributions to the $h\rightarrow \gamma \gamma$ rate do not exceed the 10–15 % level (with a sign being the same as the sign of $\mu$) [157, 158].

Of course, different contributions can sum up resulting in more sizeable shifts. However, a 50 % deviation of the rate is unlikely and occurs only in extreme situations.

### Invisible Higgs decays?

**Fig. 13:**
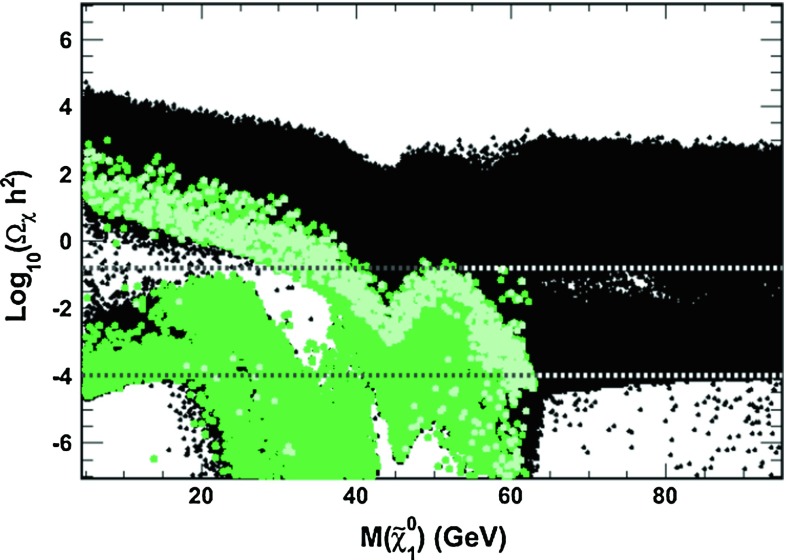
The neutralino relic density $\log _{10} (\varOmega _{\chi } h^2)$ as a function of $m_{\chi _1^0}$ compatible with BR$(h\rightarrow \chi _1^0 \chi _1^0) \ge 15\,\%$ (*green*) and the LHC Higgs data at 90 % CL (*light green*). The *horizontal lines* show the WMAP constraint on $\varOmega _{\chi } h^2$. From Ref. [149]

Invisible decays can also affect the properties of the observed $h$ particle. In the MSSM, because of the LEP2 constraints, the only possible invisible channel for the $h$ boson is into pairs of the LSP neutralinos, $h \rightarrow \chi ^0_1 \chi ^0_1$. BR$_\mathrm{inv}$ can be important for $m_{\tilde{\chi }^0_1} <$ 60GeV and for not too large $M_1$ and $|\mu |$ values which make the LSP a higgsino–gaugino mixture with significant couplings to the $h$ state. Such an LSP would have the relic density $\varOmega h^2$ required by the WMAP results [95] since it will annihilate efficiently through the s-channel exchange of the $h$ boson. However, BR$_\mathrm{inv}$ should be small in this case. This is exemplified in Fig. 13, where $\log _{10} (\varOmega _{\chi } h^2)$ is shown as a function of $m_{\chi _1^0}$ for the pMSSM points that satisfy the LHC Higgs constraints and BR$(h \rightarrow \chi _1^0 \chi _1^0) \ge 15\,\%$. Only a small area in the region $30 \lesssim m_{\chi _1^0} \lesssim 60$ GeV fulfils these conditions.

**Fig. 14:**
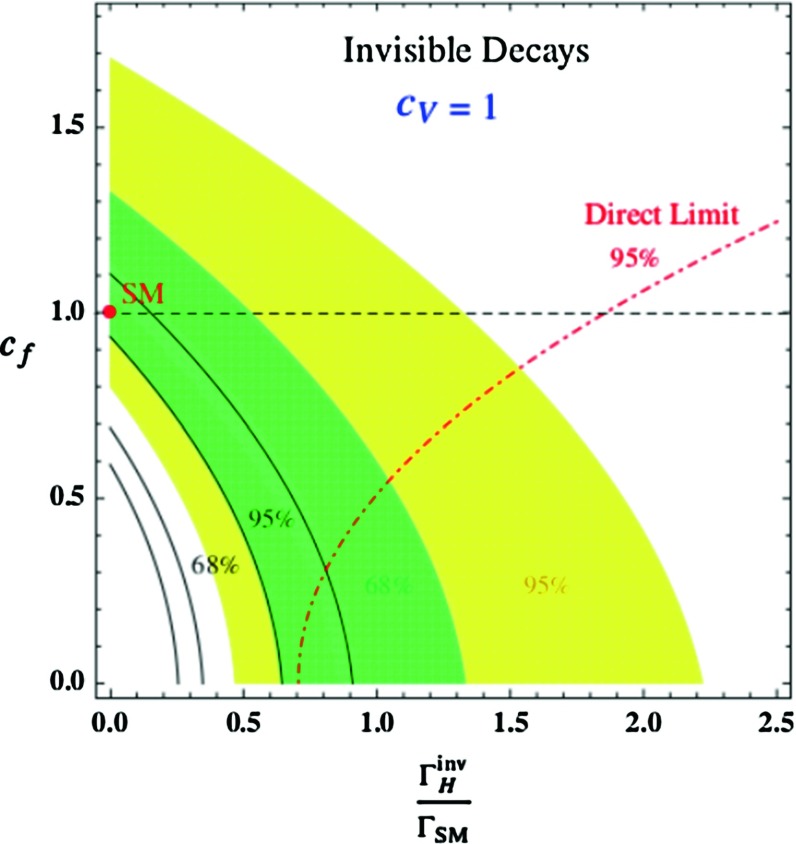
$1\sigma$ and $2\sigma$ domains from $\mu _{ZZ}$ for $c_V=1$ in the plane $[c_f, \varGamma _H^\mathrm{inv}/\varGamma _H^\mathrm{tot}]$ [150]. The dependence on the theory uncertainties are shown by the *black curves* that indicate the other possible extreme domains. The direct upper limit on $\varGamma _H^\mathrm{inv}$ from direct searches at LHC for $c_V=c_f=1$ [159] is also shown

The invisible Higgs decay width can be constrained indirectly by a fit of the Higgs couplings and in particular with the signal strength $\mu _{ZZ}$, which is the most accurate one and has the least theoretical ambiguities. $\varGamma _H^\mathrm{inv}$ enters in the signal strength through the total width $\varGamma _H^\mathrm{tot}$, $\mu _{ZZ} \propto \varGamma (H \rightarrow ZZ)/\varGamma _H^\mathrm{tot}$ with $\varGamma _H^\mathrm{tot} = \varGamma _H^\mathrm{inv} + \varGamma _H^\mathrm{SM}$ and $\varGamma _H^\mathrm{SM}$ calculated with free coefficients $c_f$ and $c_V$. The resulting $1\sigma$ or $2\sigma$ ranges are shown in Fig. 14, where $c_f$ is freely varied while $c_V=1$ [150]. This gives $\varGamma _H^\mathrm{inv}/ \varGamma _H^\mathrm{SM} \lesssim 50\,\%$ at the $95\,\%\, \mathrm{CL}$ if the assumption $c_f=c_V=1$ is made.

**Fig. 15:**
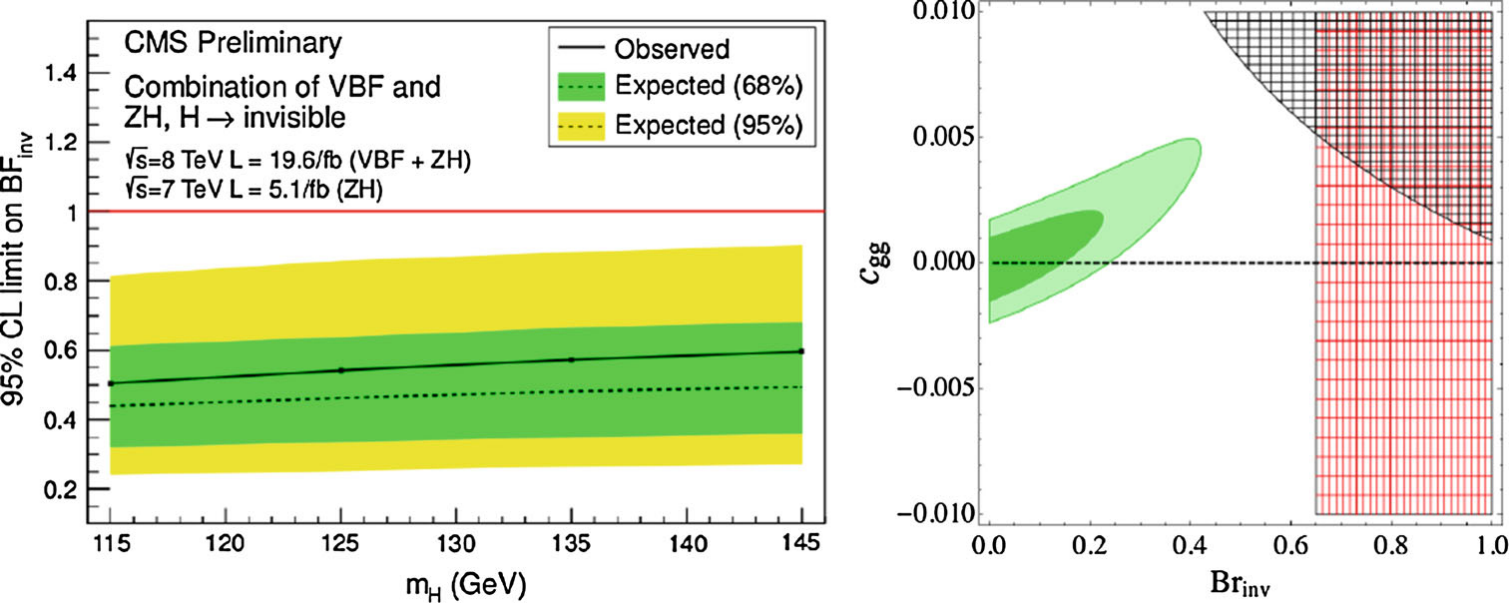
*Left:* the Higgs cross section times invisible Higgs decay branching ratio normalised to the total SM cross section in the combined $hV$ and VBF channels from CMS with the $\approx 20$ fb$^{-1}$ data at 8 TeV [163]. *Right*: $68\,\%$ CL (*light green*) and $95\,\%$ CL (*dark green*) best-fit regions to the combined LHC Higgs data. The *black region* is excluded by the monojet constraints while the *red region* is excluded by the ATLAS $Z+E{/}_{ T}$ search [159]; from Ref. [160]

A more model independent approach would be to perform direct searches for missing transverse energy. These have been conducted by ATLAS [159] and CMS [163] in the $pp\rightarrow hV$ process with $V \rightarrow jj, \ell \ell$ and in the VBF channel, $qq \rightarrow qq E_T\slash$ . As an example, we show in Fig. 15 (left) the CMS results for the Higgs cross section times BR$_\mathrm{inv}$ versus $M_h$ when the two channels are combined. For $M_h \approx 125$ GeV a bound BR$_\mathrm{inv} \lesssim 50\,\%$ is obtained at the 95 % CL.

A more promising search for invisible decays is the monojet channel. In the ggF mode, an additional jet can be emitted at NLO leading to $gg\rightarrow hj$ final states and, because the QCD corrections are large, $\sigma (H+1j$) is not much smaller than $\sigma (h+0j$). The NNLO corrections besides significantly increasing the $h+0j$ and $h+1j$ rates, lead to $h+2j$ events that also occur in VBF and VH. Hence, if the Higgs is coupled to invisible particles, it may recoil against hard QCD radiation, leading to monojet events.

In Refs. [160–162], it has been shown that the monojet signature carries a good potential to constrain the invisible decay width of a $\approx 125$ GeV Higgs boson. In a model independent fashion, constraints can be placed on the rates

22 $$\begin{aligned} R_\mathrm{inv}^\mathrm{ggF} = {\sigma (g g \rightarrow h) \times \mathrm{BR} (h \rightarrow \mathrm{inv.})\over \sigma (g g \rightarrow h)_\mathrm{SM} } \end{aligned}$$

Recent monojet searches made by CMS and ATLAS [164, 165] are sensitive to $R_\mathrm{inv}$ close to unity. This is shown in Fig 15 (right) where the best-fit region to the LHC Higgs data is displayed in the $\mathrm{Br}_\mathrm{inv}$–$c_{gg}$ plane, where $c_{gg}$ is the deviation of $\sigma (gg\rightarrow h)$ from the SM expectation [160]. For the SM value $c_{gg}=0$, $\mathrm{Br}_\mathrm{inv} \gtrsim 20\,\%$ is disfavoured at $95\,\%$ CL while for $c_{gg} > 0$, a larger rate is allowed, up to $\mathrm{Br}_\mathrm{inv} \sim 50\,\%$.

The Higgs invisible rate and the dark matter detection rate in direct astrophysical searches are correlated in Higgs-portal models. Considering the generic cases of scalar, fermionic and vectorial dark matter particles $\chi$ that couple only to the Higgs, one can translate in each case the LHC constraint $\mathrm{BR} (h \rightarrow \mathrm{inv.})$ into a constraint on the Higgs couplings to the $\chi$ particles. It turns out that these constraints are competitive with those derived from the bounds on the dark matter scattering cross section on nucleons from the best experiment so far, XENON [95].

**Fig. 16:**
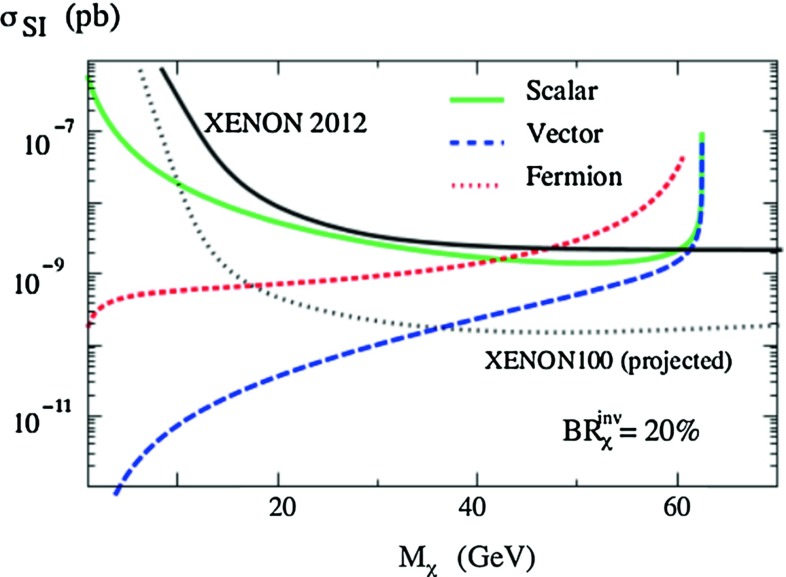
Bounds on the spin-independent direct detection cross section $\sigma ^\mathrm{SI}_{\chi p}$ in Higgs-portal models derived for an invisible branching fraction of 20 % (*colored lines*) for a 125 GeV Higgs. These are compared to the current and future direct bounds from the XENON experiment (*black lines*). From Ref. [166]

This is shown in Fig. 16, where the maximum allowed values of the scattering cross sections are given in the three cases assuming $\mathrm{BR}^\mathrm{inv}_\chi \lesssim 20\,\%$. The obtained spin-independent rates $\sigma ^\mathrm{SI}_{\chi p}$ are stronger than the direct limit from the XENON100 experiment in the entire $M_\chi \ll \frac{1}{2} M_h$ range. In other words, the LHC is currently the most sensitive dark matter detection apparatus, at least in the context of simple Higgs-portal models.

### Determination of the Higgs parity

Apart from the measurement of the couplings, one also needs in principle to establish that the observed Higgs state is indeed a CP even scalar particle and hence with $\mathrm{J^{PC}= 0^{++}}$ quantum numbers^4^. It is known that the Higgs to vector boson ($hVV$) coupling is a possible tool to probe these quantum numbers at the LHC [170, 171]. This can be done by studying various kinematical distributions in the Higgs decay and production processes. One example is the threshold behaviour of the $M_{Z^*}$ spectrum in the $h \rightarrow Z Z^* \rightarrow 4\ell$ decay channel and another is the azimuthal distribution between the decay planes of the two lepton pairs arising from the $Z, Z^*$ bosons from the Higgs decay. These are sensitive to both the spin and the parity of the Higgs.

**Fig. 17:**
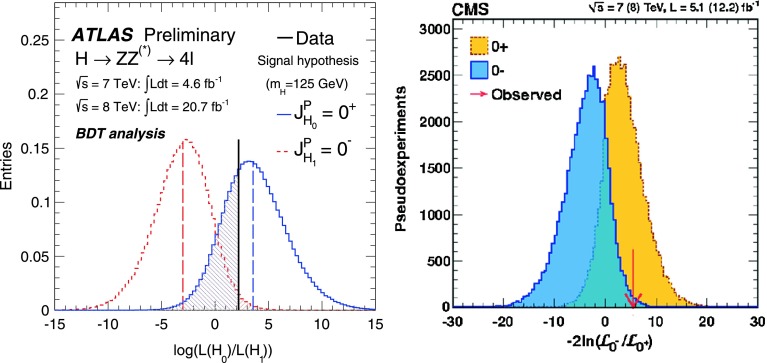
Discrimination between the $0^+$ and $0^-$ parity hypotheses for the observed Higgs boson using the kinematics of the $h\rightarrow ZZ^* \rightarrow 4\ell$ channel by the ATLAS (*left*) and CMS (*right*) collaborations with the data collected at 7+8 TeV [172, 173]

With the 25 fb$^{-1}$ data collected so far, the ATLAS and CMS collaborations performed a matrix-element likelihood analysis which exploits the kinematics and Lorenz structure of the $h\rightarrow ZZ^* \rightarrow 4\ell$ channel to see whether the angular distributions are more compatible with the $0^+$ or $0^-$ hypothesis (as well as the spin-2 possibility) [172, 173]. Assuming that it has the same couplings as the SM Higgs boson and that it is produced mainly from the dominant ggF process, the observed particle is found to be compatible with a $0^+$ state and the $0^-$ possibility is excluded at the 97.8% confidence level or higher; see Fig. 17.

Other useful diagnostics of the CP nature of the Higgs boson that also rely on the different tensorial structure of the $hVV$ coupling can be made in the VBF process. It was known since a long time that in this channel, the distribution in the azimuthal angle between the two jets produced in association with the Higgs discriminates a CP-even from a CP-odd state [174]. This has been extended recently to other observables in this process, like the rapidity separation between the two jets [175–178].

Recently, the VBF channel $pp \rightarrow Hjj$ has been reanalysed in the presence of an anomalous $hVV$ vertex that parametrises different spin and CP assignments of the produced Higgs boson [178]. The anomalous $hVV$ coupling is introduced by allowing for an effective Lagrangian with higher dimensional operators, which include four momentum terms which are absent in the SM. It was shown that the kinematics of the forward tagging jets in this process is highly sensitive to the structure of the anomalous coupling and that it can effectively discriminate between different assignments for the spin (spin-0 versus spin-2) and the parity (CP-even versus CP-odd) of the produced particle. In particular, it was found that the correlation between the separation in rapidity and the transverse momenta of the scattered quarks, in addition to the already discussed distribution of the azimuthal jet separation, can be significantly altered compared to the SM expectation.

**Fig. 18:**
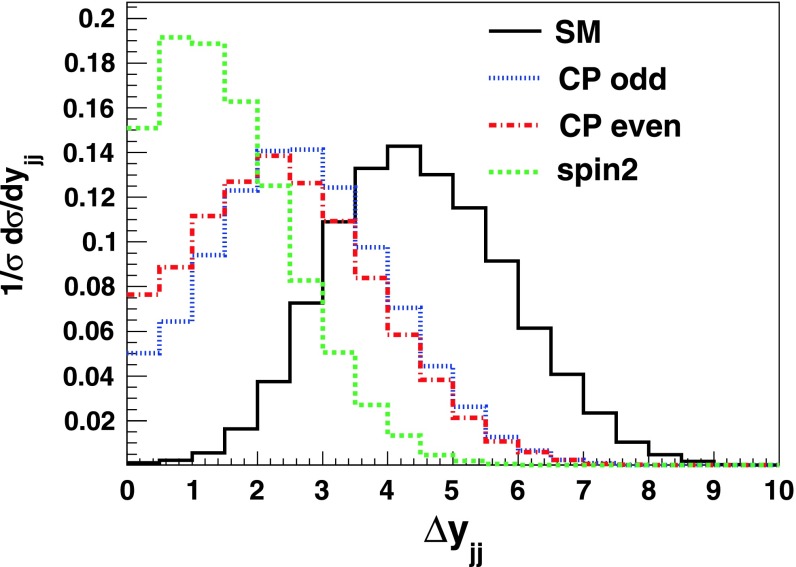
Normalised distribution of the difference in rapidity between the scattered jets in VBF for each of the SM and BSM operators (spin-2, CP-even and CP-odd) individually [178]

This is exemplified in Fig. 18, where the difference in rapidity between tagging jets ($\varDelta y_{jj}$) for each of the higher dimensional operators in the $hVV$ couplings is displayed.

These kinematical variables define new corners of the phase space that have not been explored by the experiments at the LHC to probe anomalous $hVV$ couplings and to check the Higgs parity. In addition, some of these observables significantly depend on the c.m. energy and strong constraints on anomalous couplings can be obtained by performing measurements at the LHC with energies of $\sqrt{s} = 8$ and 14 TeV. Finally, the associated $hV$ production channel can be used as the invariant mass of the $Vh$ system as well as the $p_T$ and rapidities of the $h$ and $V$ bosons are also sensitive to anomalous $hVV$ couplings.

Nevertheless, there is a caveat in the analyses relying on the $hVV$ couplings. Since a CP-odd state has no tree-level $VV$ couplings, all the previous processes project out only the CP-even component of the $hVV$ coupling [179–182] even if the state is a CP-even and -odd mixture. Thus, in the CP studies above, one is simply verifying a posteriori that indeed the CP-even component is projected out.

A better way to measure the parity of the Higgs boson is to study the signal strength in the $h\rightarrow VV$ channels [150, 183]. Indeed, the $hVV$ coupling takes the general form $g_{hVV}^{\mu \nu } = -i c_V (M_V^2/v)\; g^{\mu \nu }$ where $c_V$ measures the departure from the SM: $c_V=1$ for a pure $0^+$ state with SM-like couplings and $c_V\approx 0$ for a pure $0^-$ state. The measurement of $c_V$ should allow to determine the CP composition of the Higgs if it is indeed a mixture of $0^+$ and $0^-$ states.

However, having $c_V \ne 1$ does not automatically imply a CP-odd component: the Higgs sector can be enlarged to contain other states $h_i$ with squared $h_i VV$ couplings $\varSigma _i c_{V_i}^2\, g_{h_iVV}^2$ that reduce to the SM coupling $g_{hVV}^2$. This is what occurs in the MSSM with complex soft parameters [170, 171]: one has three neutral states $h_1, h_2$ and $h_3$ with indefinite parity and their CP-even components share the SM $hVV$ coupling, $c_{V_1}^2+c_{V_2}^2+c_{V_3}^2=1$. But in all cases, the quantity $1-c_V^2$ gives an *upper bound* on the CP-odd contribution to the $hVV$ coupling.

**Fig. 19:**
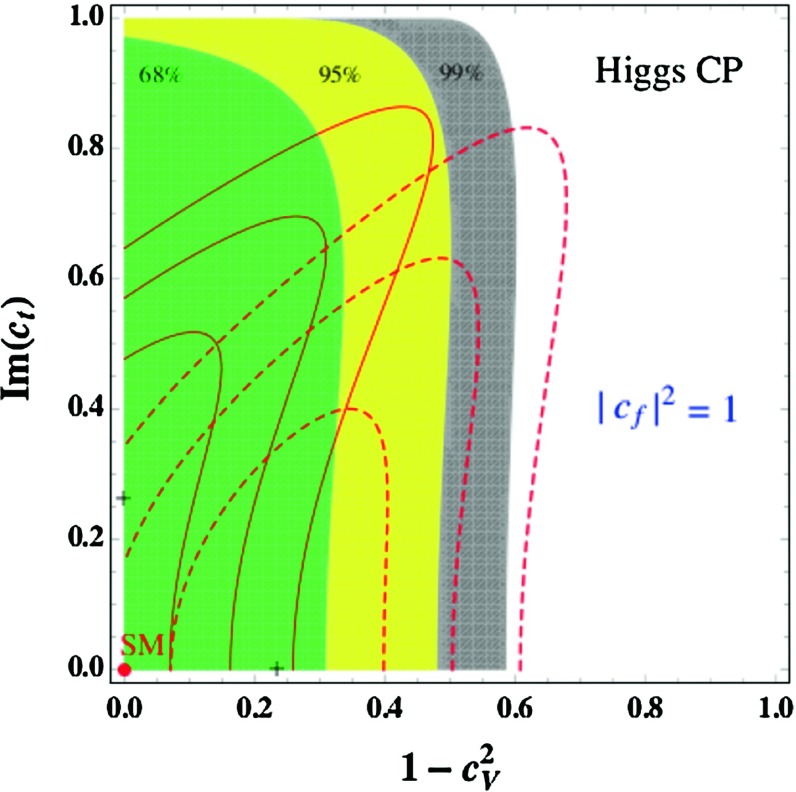
Best-fit regions at 68, 95 and $99\,\%\,\mathrm{CL}$ in the plane $[1-c^2_V$,Im$(c_t)$ for $\vert c_t \vert ^2=\vert c_f \vert ^2=1$. Superimposed are the best-fit regions when including a theory uncertainty of $\pm 20\,\%$ [150]

Using $\mu _{VV}$ and the ratios $\mu _{\gamma \gamma }/\mu _{VV}$ and $\mu _{\tau \tau }/\mu _{VV}$ as in Eq. (17), it was demonstrated that the particle has indeed a large CP component, $\gtrsim$50 % at the 95 % CL, if the Higgs couplings to fermions are SM like. This is shown in Fig. 19, where one sees that the pure CP-odd possibility is excluded at the $3 \sigma$ level, irrespective of the (mixed CP) Higgs couplings to fermions provided that $\vert c_f \vert ^2=1$.

## Implications from heavy Higgs searches

We turn now to the constraints on the MSSM Higgs sector that can be obtained from the search of the heavier $H/A$ and $H^\pm$ states at the LHC and start with a brief summary of their production and decay properties.

### $\mathbf {H,A, H^\pm }$ decays and production at the LHC

The production and decay pattern of the MSSM Higgs bosons crucially depend on $\tan \beta$. In the decoupling regime that is indicated by the $h$ properties, the heavier CP-even $H$ boson has approximately the same mass as the $A$ state and its interactions are similar. Hence, the MSSM Higgs spectrum will consist of a SM-like Higgs $h \equiv H_\mathrm{SM}$ and two pseudoscalar-like particles, $\varPhi = H/A$. The $H^\pm$ boson will also be mass degenerate with the $\varPhi$ states and the intensity of its couplings to fermions will be similar. In the high $\tan \beta$ regime, the couplings of the non-SM like Higgs bosons to $b$ quarks and to $\tau$ leptons are so strongly enhanced, and the couplings to top quarks and massive gauge bosons suppressed, that the pattern is rather simple.

This is first the case for the decays: the $\varPhi \rightarrow t\bar{t}$ channel and all other decay modes are suppressed to a level where their branching ratios are negligible and the $\varPhi$ states decay almost exclusively into $\tau ^+\tau ^-$ and $b\bar{b}$ pairs, with branching ratios of BR$(\varPhi \rightarrow \tau \tau ) \approx 10\,\%$ and BR$(\varPhi \rightarrow b \bar{b}) \approx 90\,\%$. The $H^\pm$ boson decay into $\tau \nu _{\tau }$ final states with a branching fraction of almost 100 % for $H^\pm$ masses below the $tb$ threshold, $M_{H^\pm } \lesssim m_t+m_b$, and a branching ratio of only $\approx$10 % for masses above this threshold while the rate for $H^\pm \rightarrow t b$ will be at the $\approx$90 % level in most cases.

Concerning the production, the strong enhancement of the $b$-quark couplings at high $\tan \beta$ makes that only two processes are relevant in this case: $gg \rightarrow \varPhi$ fusion with the $b$-loop included and associated production with $b$-quarks, $gg/ q\bar{q} \rightarrow b\bar{b} + \varPhi$, which is equivalent to the fusion process $b \bar{b} \rightarrow \varPhi$ when no additional final $b$-quark is present. All other processes, in particular $V\varPhi , t\bar{t} \varPhi$ and VBF have suppressed rates. In both the $b\bar{b}$ and the $gg$ fusion cases, as $M_\varPhi \gg m_b$, chiral symmetry holds and the rates are approximately the same for the CP-even $H$ and CP-odd $A$ bosons. While $\sigma (gg\rightarrow \varPhi )$ is known up to NLO in QCD [184], $\sigma (bb\rightarrow \varPhi )$ is instead known up to NNLO [185, 186].

**Fig. 20:**
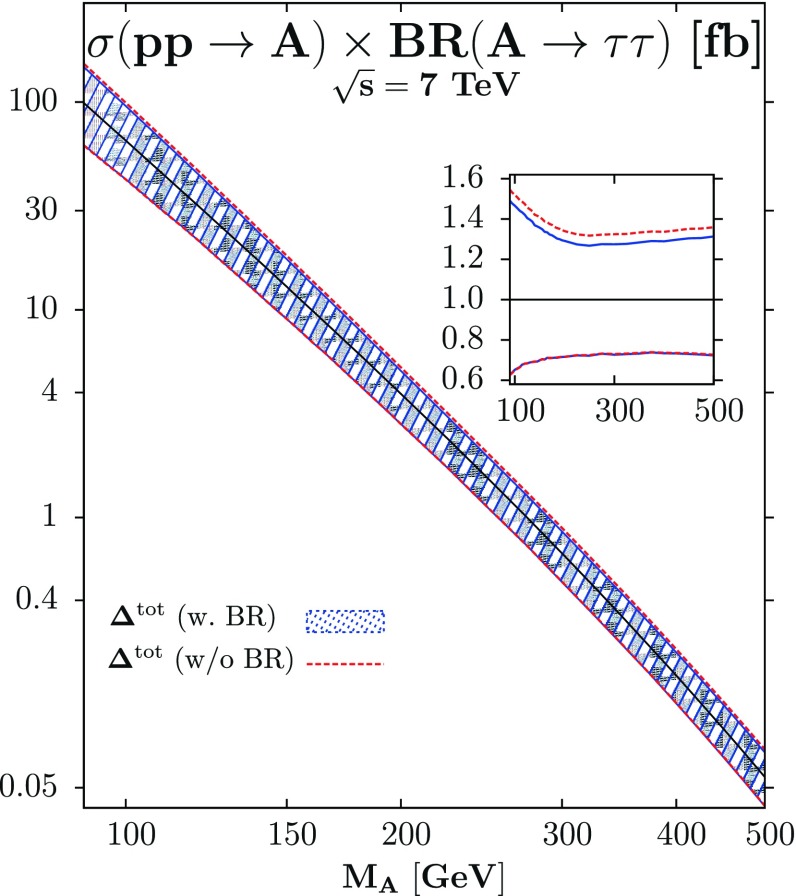
The combined $\sigma (p p \rightarrow A)\times \mathrm{BR}(A \rightarrow \tau \tau )$ rate with theoretical uncertainties with and without the branching ratio; in the *inserts*, shown are the uncertainties when the rates are normalised to the central values. From Ref. [112]

The most powerful search channel for the heavier MSSM Higgs particles at the LHC is by far the process $pp \rightarrow gg + b \bar{b} \rightarrow \varPhi \rightarrow \tau ^+ \tau ^-$. The precise values of the cross section times branching fraction for this process at the LHC have been updated in Refs. [110, 112] and an assessment of the associated theoretical uncertainties has been made. It turns out that, in the production cross section, the total uncertainty from scale variation, the PDFs and $\alpha _s$ as well as from the $b$-quark mass are not that small: $\varDelta ^\mathrm{TH} \sigma (pp\rightarrow \varPhi ) \times \mathrm{BR} (\varPhi \rightarrow \tau \tau ) \approx \pm 25\,\%$ in the entire $M_\varPhi$ range probed at the LHC at $\sqrt{s}=8$ TeV; Fig. 20.

**Fig. 21:**
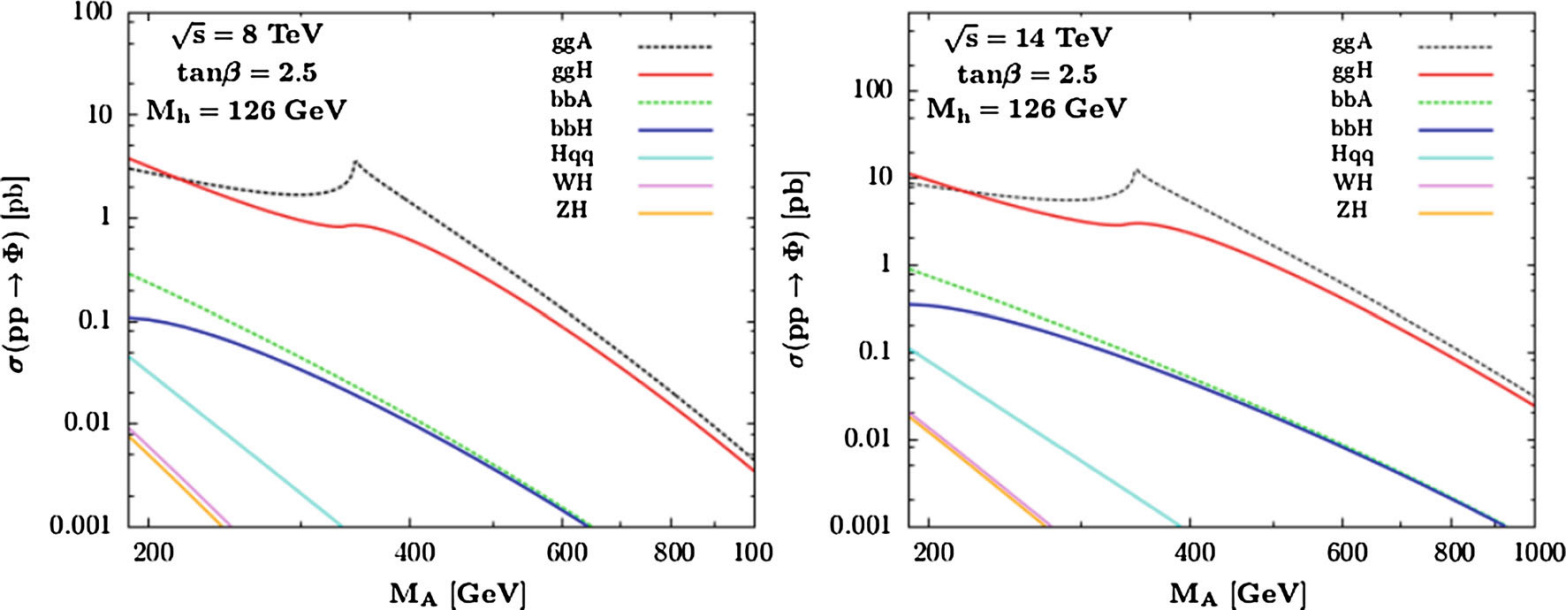
The production cross sections of the MSSM heavier neutral Higgs bosons at the LHC at $\sqrt{s}=8$ for $\tan \beta =2.5$; only the main production channels are considered [98]

Besides the QCD uncertainty, three other features could alter the rate $\sigma (pp\rightarrow \varPhi \rightarrow \tau \tau )$ in the MSSM and they are related to the impact of the SUSY particle contributions:

(i) In the case of $H$ ($A$ does not couple to identical sfermions), there are squark (mainly stop) loops that contribute in addition in the $gg\rightarrow H$ process. But as they are damped by powers of $\tilde{m}^2_Q$ for $M_H \lesssim 2m^2_Q$, these contributions should be small for $\tilde{m}_Q \gtrsim 1$ TeV, in particular at high $\tan \beta$ where the $b$-contribution is strongly enhanced.
(ii) The vertex correction to the $\varPhi b\bar{b}$ couplings, $\varDelta _b$ of Eq. (19), grows as $\mu \tan \beta$ and can be very large in the high $\tan \beta$ regime. However, in the full process $p p \rightarrow \varPhi \rightarrow \tau ^+ \tau ^-$, this correction appears in both the cross section and the branching fraction and largely cancels outs as one obtains, $\sigma \times \mathrm{BR} \times (1 - \varDelta _b/5)$. A very large contribution $\varDelta _b \approx 1$ changes the rate only by 20 %, i.e. less than the QCD uncertainty.
(iii) The possibility of light sparticles would lead to the opening of $H/A$ decays into SUSY channels that would reduce BR($\varPhi \rightarrow \tau \tau$). For $M_\varPhi \lesssim 1$ TeV, the only possibilities are decays into light neutralinos or charginos and sleptons. These are in general disfavoured at high $\tan \beta$ as the $\varPhi \rightarrow b\bar{b}+\tau \tau$ modes are strongly enhanced and dominant.

Thus, only in the unlikely cases where the decay $H \rightarrow \tilde{\tau }_1 \tilde{\tau }_1$ has a branching rate of the order of 50 %, the squark loop contribution to the $gg \rightarrow H$ process is of the order 50 %, or the $\varDelta _b$ SUSY correction is larger than 100 %, and one can change the $pp \rightarrow \varPhi \rightarrow \tau \tau$ rate by $\approx$25 %, which is the level of the QCD uncertainty. One thus expects $\sigma (pp \rightarrow \varPhi )\times \mathrm{BR}(\varPhi \rightarrow \tau \tau )$ to be extremely robust and to depend almost exclusively on $M_A$ and $\tan \beta$.

Finally, for the charged Higgs boson, the dominant search channel is in $H^\pm \rightarrow \tau \nu$ final states with the $H^\pm$ bosons produced in top quark decays for $M_{H^\pm } \lesssim m_t- m_b \approx 170$ GeV, $pp \rightarrow t\bar{t}$ with $t \rightarrow H^+ b \rightarrow \tau \nu b$. This is particularly true at high $\tan \beta$ values when BR($t\rightarrow H^+b) \propto \bar{m}_b^2 \tan ^2\beta$ is significant. For higher $H^\pm$ masses, one should rely on the three-body production process $pp \rightarrow tbH^\pm \rightarrow tb \tau \nu$, but the rates are presently rather small.

In the low $\tan \beta$ regime, $\tan \beta \lesssim 5$, the phenomenology of the heavier $A,H,H^\pm$ bosons is richer [98, 187–190]. Starting with the cross sections, we display in Fig. 21 the rates for the relevant production processes at the LHC with $\sqrt{s}=8$ TeV assuming $\tan \beta =2.5$. For smaller $\tan \beta$ values, the rates except for $pp \rightarrow H/A+b\bar{b}$ are even larger as the $H/A+ tt$ and $HVV$ couplings are less suppressed.

**Fig. 22:**
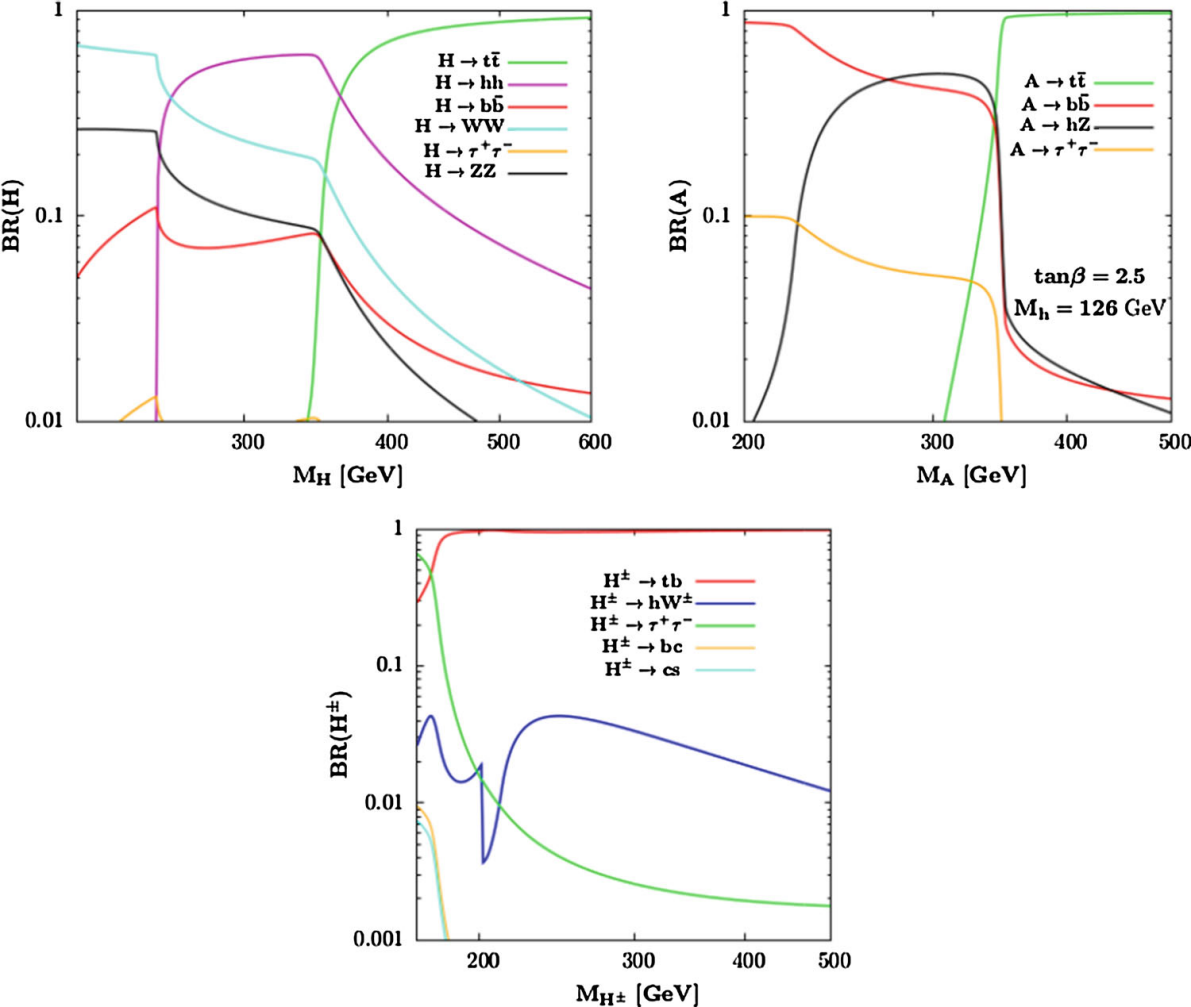
The $H/A/H^\pm$ decay branching ratios as functions of the Higgs masses for $\tan \beta =2.5$ [98]

Because of CP invariance which forbids $AVV$ couplings, there is no $AV$ and $Aqq$ processes while the rates for associated $t\bar{t} A$ and $b\bar{b} A$ are small because the $Att\;(Abb)$ couplings are suppressed (not sufficiently enhanced) compared to the SM Higgs. Only the $gg\rightarrow A$ process with the dominant $t$ and sub-dominant $b$ contributions included provides large rates. The situation is almost the same for $H$: only $gg\rightarrow H$ is significant at $M_H \gtrsim 300$ GeV and $\tan \beta \lesssim 5$; the VBF and HV modes give additional small contributions for $\tan \beta \approx 1$. For $H^\pm$, the dominant production channel is again top quark decays, $t \rightarrow H^+ b$ for $M_{H^\pm } \lesssim 170$ GeV as for $\tan \beta \lesssim 5$, the $m_t/\tan \beta$ piece of the $H^\pm tb$ coupling becomes large; for higher $H^\pm$ masses, the main process to be considered is $gg/q\bar{q} \rightarrow H^\pm tb$.

Turning to the $H/A/H^\pm$ decay pattern, it can be rather involved at low $\tan \beta$. A summary is as follows for $\tan \beta \lesssim 3$; see also Fig. 22, where the rates are shown for $\tan \beta =2.5$.

- Above the $t\bar{t}\; (tb)$ threshold for $H/A(H^\pm )$, the decay channels $H/ A \rightarrow t\bar{t}$ and $H^+ \rightarrow t \bar{b}$ are by far dominant for $\tan \beta \lesssim 3$ and do not leave space for any other mode.
- Below the $t\bar{t}$ threshold, the $H \rightarrow WW,ZZ$ decay rates are still significant as $g_{HVV}$ is not completely suppressed.
- For $2M_h \lesssim M_H \lesssim 2m_t$, $H\rightarrow hh$ is the dominant $H$ decay mode as the $Hhh$ self-coupling is large at low $\tan \beta$.
- For $M_A \gtrsim M_h+M_Z$, $A \rightarrow hZ$ decays would occur but the $A \rightarrow \tau \tau$ channel is still important with rates $\gtrsim$5 %.
- In the case of $H^\pm$, the channel $H^+ \rightarrow Wh$ is important for $M_{H^\pm } \lesssim 250$ GeV, similarly to the $A \rightarrow hZ$ case.

Hence, many decay and production channels need to be considered in this low $\tan \beta$ regime.

### Constraints from the LHC Higgs searches

The most efficient channel to probe the heavier MSSM Higgs bosons is by far $pp \rightarrow gg + bb \rightarrow H/A \rightarrow \tau ^+ \tau ^-$. Searches for this process have been performed by ATLAS with $\approx$5 fb$^{-1}$ data at the 7 TeV run [191] and by CMS with $\approx$5+12 fb$^{-1}$ data at the 7 and 8 TeV runs [192]. Upper limits on the production cross section times decay branching ratio have been set and they can be turned into constraints on the MSSM parameter space.

**Fig. 23:**
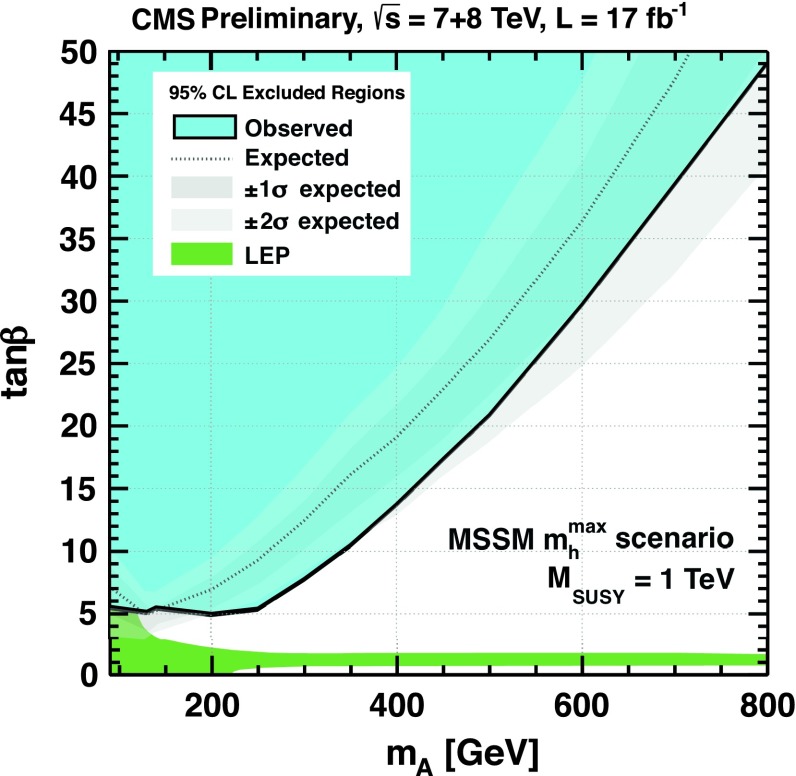
The expected and observed exclusion limits in the $[\tan \beta , M_A]$ plane in the CMS search of the MSSM neutral Higgs bosons in the channels $pp \rightarrow h/H/A \rightarrow \tau ^+\tau ^-$ with $\approx 17$ fb$^{-1}$ data collected at $\sqrt{s}=7$+8 TeV [192]

In Fig. 23, the sensitivity is displayed of the CMS $pp\rightarrow \varPhi \rightarrow \tau \tau$ analysis with 17 fb$^{-1}$ of data in the $[\tan \beta ,M_A]$ plane. The excluded region, obtained from the observed limit at the 95 % CL, is drawn in blue. The dotted line represents the median expected limit which turns out to be weaker than the observed limit. As can be seen, this constraint is extremely restrictive and, for values $M_A \lesssim 250$ GeV, it excludes almost the entire intermediate and high $\tan \beta$ regimes, $\tan \beta \gtrsim 5$. The constraint is less effective for a heavier $A$ boson, but even for $M_A \approx 400$ GeV the high $\tan \beta \gtrsim 10$ region is excluded and there is even sensitivity to large values $M_A \approx 800$ GeV for $\tan \beta \gtrsim 50$.

There are, however, some caveats to this exclusion limit as discussed previously. The first one is that there is a theoretical uncertainty of order of $\pm 25\,\%$ that affects the $gg \rightarrow \varPhi$ and $b\bar{b} \rightarrow \varPhi$ production cross sections which, when included, will make the constraint slightly weaker as one then needs to consider the lower value predicted for the production rate. A second caveat is that SUSY effects, direct corrections to the production and $H/A$ decays into sparticles, could alter the rate. However, as previously argued, $\sigma (pp \rightarrow \varPhi ) \times \mathrm{BR}(\varPhi \rightarrow \tau \tau )$ is robust against these SUSY effects and the latter will unlikely make a substantial change of the cross section times branching fraction. Finally, the constraint is specifically given in the maximal mixing scenario $X_t/M_S= \sqrt{6}$ with $M_S=1$ TeV. The robustness of $\sigma \times \mathrm{BR}$ makes that the exclusion limit is actually almost model independent and is valid in far more situations than the “MSSM $M_h^\mathrm{max}$ scenario” quoted there, an assumption that can be removed without any loss.

In fact, the exclusion limit can also be extended to the low $\tan \beta$ region which, in the chosen scenario with $M_S=1$ TeV, is excluded by the LEP2 limit on the lighter $h$ mass (the green area in the figure) but should resurrect if the SUSY scale is kept as a free parameter. Note also that $H/A$ bosons have also been searched for in the channel $gg \rightarrow b\bar{b} \varPhi$ with $\varPhi \rightarrow b\bar{b}$ (requiring more than three tagged $b$ jets in the final state) but the constraints are much less severe than the ones derived from the $\tau \tau$ channel [193].

**Fig. 24:**
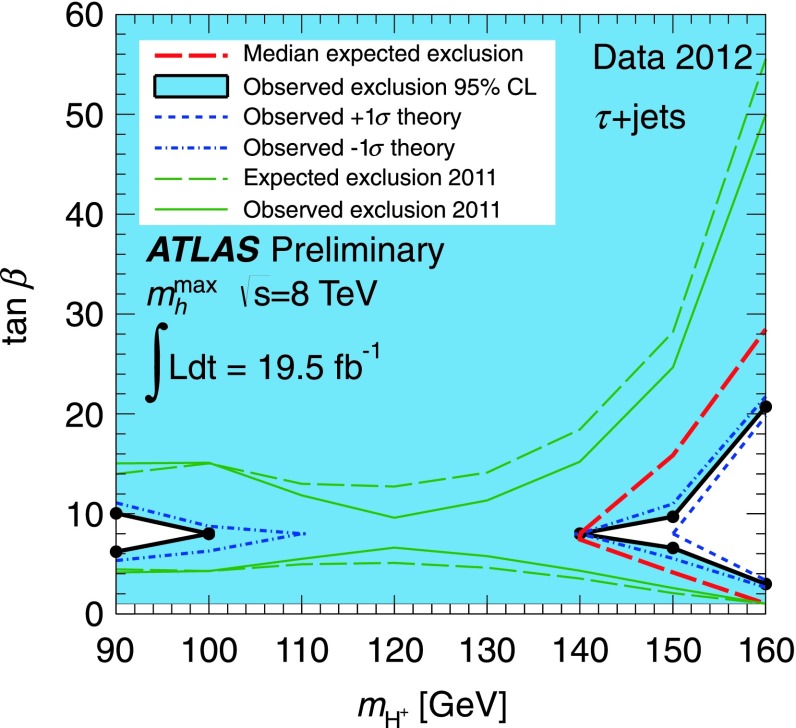
The $H^\pm$ limits from ATLAS with $\sqrt{s}=8$ TeV and $\approx$20 fb$^{-1}$ data in the channel $t\rightarrow bH^+ \rightarrow b\tau \nu$ [194]

Turning to the $H^+$ boson [194, 195], the most recent result has been provided by the ATLAS collaboration using the full $\approx$20 fb$^{-1}$ data collected at $\sqrt{s}= 8$ TeV. The $H^\pm$ search as been performed using the $\tau$ plus jets channel with a hadronically decaying $\tau$ lepton in the final state. For $M_{H^\pm } \lesssim 160$ GeV, the results are shown in Fig. 24. Here, the relevant process is top quark decays, $t \rightarrow H^+ b$ with the decay $H^+ \rightarrow \tau \nu$ having a branching ratio of almost 100 % at moderate to high $\tan \beta$. For these high values, the $H^+tb$ coupling has a component $\propto m_b \tan \beta$, which makes BR($t \rightarrow _!H^+b)$ rather large. Almost the entire $\tan \beta \gtrsim 10$ region is excluded by the ATLAS analysis.

In addition, the branching fraction for the decay $t \rightarrow bH^+$ is also significant at low $\tan \beta$ values, when the component of the coupling $g_{tbH^+} \propto \bar{m}_t /\tan \beta$ becomes dominant. On the other hand, the branching fraction for the decay $H^\pm \rightarrow \tau \nu$ does not become very small as it has competition only from $H^+ \rightarrow c\bar{s}$, which, even for $\tan \beta \approx 1$, does not dominate. Hence, the rates for $pp \rightarrow t\bar{t}$ with $t \rightarrow bH^+ \rightarrow b\tau \nu$ are comparable for $\tan \beta \approx 3$ and $\tan \beta \approx 30$ and the processes can also probe the low $\tan \beta$ region. This is exemplified in Fig. 24 where one can see that the entire area below $\tan \beta \approx 5$ is also excluded. There remains then, for $H^\pm$ masses close to 90 GeV (where the detection efficiency is lower) and 160 GeV (where one is limited by the phase space), the intermediate area with $\tan \beta \approx 5$–10 where the $H^\pm tb$ coupling is not strongly enhanced.

This ATLAS search has been extended to larger values of $M_{H^\pm }$ where the charged Higgs is produced in association with top quarks, $gb \rightarrow tH^+$, but the constraints are poor (only the region $\tan \beta \gtrsim 50$ is excluded for $M_{H^\pm } =200$–300 GeV) as the cross section for this process is low.

The reopening of the low $\tan \beta$ region allows to consider a plethora of very interesting channels for the heavier Higgs bosons to be also investigated at the LHC: heavier CP-even $H$ decays into massive gauge bosons $H\rightarrow WW,ZZ$ and Higgs bosons $H\rightarrow hh$, CP-odd Higgs decays into a vector and a Higgs boson, $A \rightarrow hZ$, CP-even and CP-odd Higgs decays into top quarks, $H/A \rightarrow t \bar{t}$, and even the charged Higgs decay $H^\pm \rightarrow Wh$. These final states have been searched for in the context of a heavy SM Higgs boson or for new resonances in some non-SUSY beyond the SM scenarios and the analyses can be adapted to the case of the heavier MSSM Higgs bosons. They would then allow to cover a larger part of the parameter space of the MSSM Higgs sector in a model-independent way, i.e. without using the information on the scale $M_S$ and more generally on the SUSY particle spectrum that appear in the radiative corrections.

**Fig. 25:**
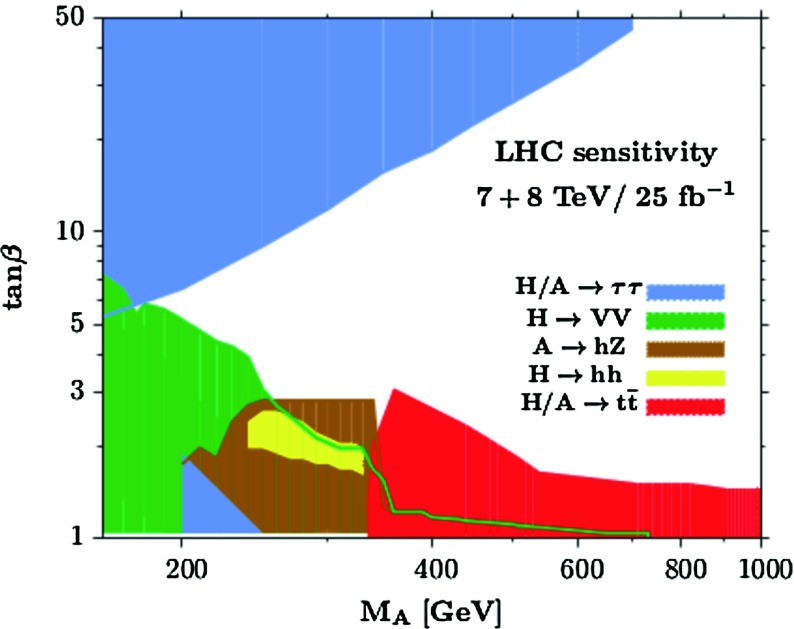
The estimated sensitivities in the various search channels for the heavier MSSM Higgs bosons in the $[\tan \beta ,M_A]$ plane: $H/A \rightarrow \tau \tau$, $H \rightarrow WW + ZZ$, $H/A \rightarrow t\bar{t}$, $A \rightarrow hZ$ and $H \rightarrow hh$ [98]. The projection is made for the LHC with 7+8 TeV and the full 25 fb$^{-1}$ of data collected so far. The radiative corrections are such that the $h$ mass is $M_h=126$ GeV

In Ref. [98] a preliminary analysis of these channels has been performed using current information given by the ATLAS and CMS collaborations in the context of searches for the SM Higgs boson or other heavy resonances (in particular new $Z'$ or Kaluza–Klein gauge bosons that decay into $t\bar{t}$ pairs). The results are shown in Fig. 25 with an extrapolation to the full 25 fb$^{-1}$ data of the 7+8 TeV LHC run (it has been assumed that the sensitivity scales simply as the square root of the number of events). The sensitivities from the usual $H/A \rightarrow \tau ^+\tau ^-$ and $t \rightarrow bH^+ \rightarrow b \tau \nu$ channels are also shown. The green and red areas correspond to the domains where the $H\rightarrow VV$ and $H/A \rightarrow t\bar{t}$ channels become constraining. The sensitivities in the $H\rightarrow hh$ and $A\rightarrow hZ$ modes are given by, respectively, the yellow and brown areas which peak in the mass range $M_A=250$–350 GeV that is visible at low $\tan \beta$ values.

The outcome is impressive. These channels, in particular the $H \rightarrow VV$ and $H/A \rightarrow t \bar{t}$ processes, are very constraining as they cover the entire low $\tan \beta$ area that was previously excluded by the LEP2 bound up to $M_A \approx 500$ GeV. Even $A \rightarrow hZ$ and $H \rightarrow hh$ would be visible at the current LHC in small portions of the parameter space.

### Could the observed state be the heavier H boson?

Let us briefly discuss the possibility, raised with the early LHC data, that the observed particle is the heavier MSSM $H$ boson, as advocated for instance in Refs. [60–62, 105]. The possibility $M_H \approx 125$ GeV, with $H$ couplings close to those of the SM Higgs, occurs for low values of $M_A$, $\approx$100–120 GeV, and moderate values of $\tan \beta$, $\approx 10$. In this case, $H$ has approximately SM-like properties, while $h$ has a mass of order 100 GeV or below and suppressed couplings to vector bosons. A dedicated scan for this region of parameter space has been performed [36] and the results were confronted with the measured Higgs mass $M_h =123$–129 GeV and couplings that comply with the LHC $\approx 10$ fb$^{-1}$ data collected at $\sqrt{s} =$ 7+8 TeV. Both the signal strengths in the various search channels of the observed Higgs boson and the limits from the $pp \rightarrow \tau ^+ \tau ^-$ channel obtained by the CMS collaboration have been considered.

It was found that among the large flat scan with $10^8$ points, only $\approx 2 \times 10^{-5}$ of the generated points would remain after imposing these LHC constraints. These points were then excluded by applying the constraints from flavour physics [94] (see also Ref. [196]), mainly the radiative decay $b\rightarrow s\gamma$ and dark matter constraints [95] (as they do not satisfy the constraint of $10^{-4} < \varOmega h^2 < 0.155$ when accounting for all uncertainties). The updated $pp\rightarrow \tau ^+\tau ^-$ search performed by CMS with 17 fb$^{-1}$ data, which excludes all values $\tan \beta \gtrsim 5$ for $M_A \lesssim 250$ GeV as shown in Fig. 23, now definitely rules out this scenario.

**Fig. 26:**
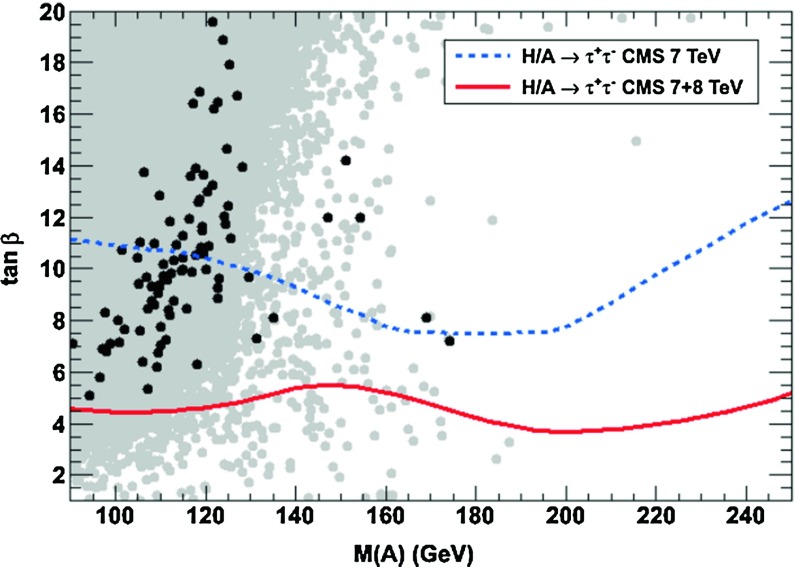
The parameter space $[M_A, \tan \beta ]$ with points for the heavier $H$ boson to be observed with a mass in the range 123–129 GeV (*light grey points*) and after flavour and dark matter relic density constraints (*black points*) [36]. The CMS excluded regions from the 2011 and 2012 $\varPhi \rightarrow \tau ^+ \tau ^-$ searches are shown by the *dashed blue* and continuous *red lines*, respectively

This is exemplified in Fig. 26, where we zoom in the $[M_A, \tan \beta ]$ plane for low values of the inputs and apply the constraints listed above. The small region in which the $H$ boson was allowed to be the observed state (black points) by the previous $H/A \rightarrow \tau ^+ \tau ^-$ CMS search (dashed blue line) is excluded by the new data (in red). In fact, the latest ATLAS limits from $H^\pm$ searches given in Fig. 24 also exclude now the possibility $M_A\approx 100$–120 GeV and, hence, the scenario where $H$ is the observed Higgs state^5^.

### Higgs production with SUSY particles

Finally, let us comment on the possibility of the Higgs bosons being produced in processes involving sparticles. First of all, there is the option of Higgs decays into SUSY particles. In the case of the lighter $h$ boson, the only possibility when the LEP2 constraints are taken into account is the decay $h \rightarrow \chi _1^0 \chi _1^0$, which has been discussed in the context of invisible Higgs decays in Sect. 3.4. In view of the strong LHC limits on squark masses, the only SUSY channels of the heavier $H/A/H^\pm$ states that might be kinematically open would be the decays into chargino, neutralinos and sleptons. For $H/A$, these decays have been discussed in the context of the $\tau$ searches as they might reduce the $H/A \rightarrow \tau \tau$ branching fractions but no specific search for these SUSY final states has been performed so far.

Turning to associated Higgs production with sparticles, the most important process was expected to be $pp \rightarrow \tilde{t}_1 \tilde{t}_1+$ Higgs which could benefit from the possibly large Higgs–stop coupling [199–202]. The large value of $M_S$ and hence the lightest stop mass from the current constraint makes this process unlikely. Another possibility would be associated production with staus where the phase space could be more favourable but the rates are in general much smaller.

The only channel which could lead to a detectable signal with the data collected so far would be Higgs particles from decays of charginos and neutralinos. In particular the decays $\chi _2^0 \rightarrow \chi _1^0 h$, with $\chi _2^0$ directly produced in association with $\chi _1^\pm$ in the process $pp \rightarrow \chi _2^0 \chi _1^\pm$ leading a lepton, a Higgs (decaying either into $b\bar{b}$ or into multi-leptons via $h\rightarrow ZZ^*, WW^*$) and missing energy [203–208].

**Fig. 27:**
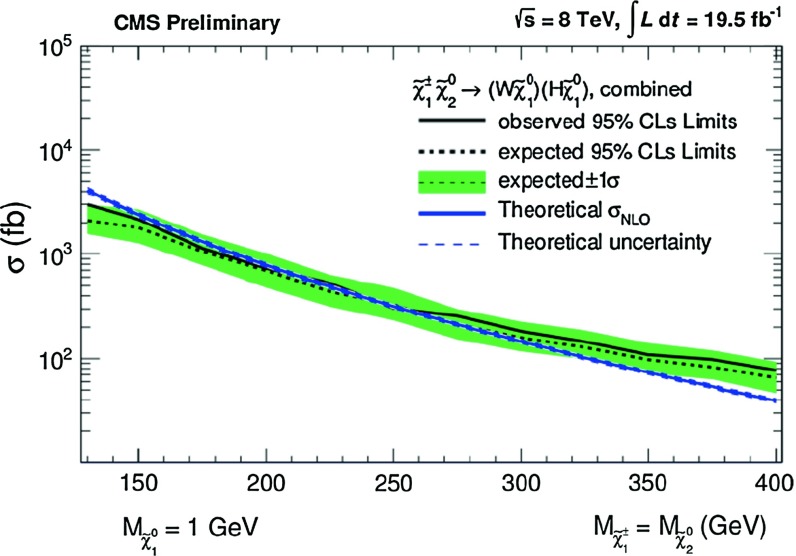
The interpretations of the CMS results from the combination of all lepton and $E{/}_T$ searches with $\approx$20 fb$^{-1}$ data collected at $\sqrt{s}=8$ TeV. The expected and observed limits on the $pp\rightarrow \chi _2^0 \chi _1^\pm$ cross section times the $\chi _2^0 \chi _1^\pm \rightarrow Wh \chi _1^0 \chi _1^0$ branching fraction (with the *green* band is for experimental uncertainties) is compared to the theoretical prediction [209]

The CMS collaboration has reported the results for searches of leptons and missing energy with a luminosity of $\approx$20 fb$^{-1}$ data collected at $\sqrt{s}=8$ TeV [209]. They set a limit on the cross section times branching ratio for the possible SUSY process $pp\rightarrow \chi _2^0 \chi _1^\pm$ with $\chi _2^0 \rightarrow \chi _1^0 h$ and $\chi _1^\pm \rightarrow W \chi _1^0$. As can be observed from Fig. 27, where the cross section times branching ratio is displayed as a function of the masses $m_{\chi _1^\pm }=m_{\chi _2^0}$ (with the assumption that the LSP neutralino is very light, $m_{\chi _1^0}=1$ GeV), the data show no excess over the SM backgrounds.

## What next?

The last 2 years were extremely rich and exciting for particle physics. With the historical discovery at the LHC of a Higgs boson by the ATLAS and CMS collaboration crowned by a Nobel price this fall, and the first probe of its basic properties, they witnessed a giant step in the unravelling of the mechanism that breaks the electroweak symmetry and generates the fundamental particle masses. They promoted the SM as the appropriate theory, up to at least the Fermi energy scale, to describe three of Nature’s interactions, the electromagnetic, weak and strong forces,

However, it is clear that these 2 years have also led to some frustration, as no signal of physics beyond the SM has emerged from the LHC data. The hope of observing some signs of the new physics models that were put forward to address the hierarchy problem, which is deeply rooted in the Higgs mechanisms, with supersymmetric theories being the most attractive ones, did not materialise.

The discovery of the Higgs boson and the non-observation of new particles has nevertheless far reaching consequences for supersymmetric theories and, in particular, for their simplest low-energy formulation, the MSSM. The mass of approximately 125 GeV of the observed Higgs boson implies that the scale of SUSY-breaking is rather high, at least ${\mathcal {O}}$(TeV). This is backed up by the limits on the masses of strongly interacting SUSY particles set by the ATLAS and CMS searches, which in most cases exceed the TeV range [86, 87]. This implies that if SUSY is indeed behind the stabilisation of the Higgs mass against very high scales that enter via quantum corrections, it is either fine-tuned at the permille level at least or its low-energy manifestation is more complicated than expected.

The production and decay rates of the observed Higgs particles, as well as its spin and parity quantum numbers, as measured by the ATLAS and CMS collaborations with the $\approx$ 25 fb$^{-1}$ data collected at $\sqrt{s}=7$ and 8 TeV, indicate that its couplings to fermions and gauge bosons are approximately SM-like. In the context of the MSSM, this implies that we seem to be in the decoupling regime and this 125 GeV particle can be only identified with the lightest $h$ boson, while the other $H/A/H^\pm$ states must be heavier than approximately the Fermi scale. This last feature is also backed up by the constraints from direct searches of these heavier Higgs states at the LHC.

This drives up to the question that is now very often asked in particle physics (and elsewhere): what to do next? The answer is, for me, obvious: we are only in the beginning of a new era.^6^ Indeed, it was expected since a long time that the probing of the EWSB mechanism will be at least a two chapters story. The first one is the search and the observation of a Higgs-like particle that will confirm the scenario of the SM and most of its extensions, that is, a spontaneous symmetry breaking by a scalar field that develops a non-zero vacuum expectation value. This long chapter has just been closed by the ATLAS and CMS collaborations with the spectacular observation of a Higgs boson. This observation opens a second and equally important chapter: the precise determination of the Higgs profile and the unravelling of the EWSB mechanism itself.

A more accurate measurement of the Higgs couplings to fermions and gauge bosons will be mandatory to establish the exact nature of the mechanism and, eventually, to pin down effects of new physics if additional ingredients beyond those of the SM are involved. This is particularly true in weakly interacting theories such as SUSY in which the quantum effects are expected to be small. These measurements could be performed at the upgraded LHC with an energy close to $\sqrt{s}=14$ TeV, in particular if a very high luminosity, a few ab$^{-1}$, is achieved [210–212].

At this upgrade, besides improving the measurements performed so far, rare but important channels such as associated Higgs production with top quarks, $pp\rightarrow t\bar{t} h$, and Higgs decays into $\mu ^+ \mu ^-$ and $Z\gamma$ states could be probed. Above all, a determination of the self-Higgs coupling could be made by searching for double Higgs production e.g. in the gluon fusion channel $gg\rightarrow hh$ [213–215]; this would be a first step towards the reconstruction of the scalar potential that is responsible of EWSB. A proton collider with an energy $\sqrt{s}=30$ to 100 TeV could do a similar job [212].

In a less near future, a high-energy lepton collider, which is nowadays discussed in various options (ILC, TLEP, CLIC, $\mu$ collider) would lead to a more accurate probing of the Higgs properties [216–224], promoting the scalar sector of the theory to the high-precision level of the gauge and fermionic sectors achieved by LEP and SLC [30].

Besides the high precision study of the already observed Higgs, one should also continue to search for the heavy states that are predicted by SUSY, not only the superparticles but also the heavier Higgs bosons. The energy upgrade to $\approx$14 TeV (and eventually beyond) and the planed order of magnitude (or more) increase in luminosity will allow to probe much higher mass scales than presently.

In conclusion, it is not yet time to give up on supersymmetry and on new physics in general but, rather, to work harder to be fully prepared for the more precise and larger data that will be delivered by the upgraded LHC. It will be soon enough to “philosophise” in 2 years from now, when the physics landscape will become clearer.

## Footnotes

1 This uncertainty is obtained by comparing the outputs of SuSpect and FeynHiggs [83] which use different schemes for the radiative corrections: while the former uses the $\overline{\mathrm{DR}}$ scheme, the latter uses the on-shell scheme; the difference in the obtained $M_h$ amounts to $\approx \pm 2$–3 GeV in general. To this, one has to add an uncertainty of $\pm 1$ GeV from the top quark mass measurement at the Tevatron, $m_t=173 \pm 1$ GeV [85]. Note that it is not entirely clear whether this mass is indeed the pole quark mass. A more rigorous determination of the pole mass from the measured top-pair cross section at the Tevatron gives a lower value with a larger uncertainty, $m_t^\mathrm{pole} =171 \pm 3$ GeV [84].

2 Note that also in the VBF process, despite the fact that the inclusive cross section has only a few percent combined scale and PDF uncertainty [110], the contamination by the $gg \rightarrow h+2j$ channel makes the total uncertainty in the $h+jj$ final “VBF” sample rather large. Indeed ${\mathcal {O}} (30\,\%)$ $gg \rightarrow h+2j$ events will remain even after the specific cuts that select the VBF configuration have been applied, and the rate is affected by a much larger uncertainty than the inclusive $gg\rightarrow h$ process, up to 50 % when one adds the scale and PDF uncertainties [113].

3 In the case of the production process $gg/q\bar{q} \rightarrow h t\bar{t}$, it is still $c_t^0$, which should describe the $ht\bar{t}$ coupling, but the constraints on the $h$ properties from this process are presently very weak.

4 To be more general, the spin of the particle needs also to be determined. The observation of the $h\rightarrow \gamma \gamma$ decay channel rules out the spin-1 case by virtue of the Landau–Yang theorem [167, 168] and leaves only the spin 0 and $\ge$2 possibilities. The graviton-like spin-2 option is extremely unlikely and, already from the particle signal strengths in the various channels, it is ruled out in large classes of beyond the SM models; see e.g. Ref. [169].

5 Note that the recent $pp\rightarrow \tau \tau$ and $H^+\rightarrow \tau \nu$ limits also exclude the so-called “intense coupling regime” [197, 198], in which the three neutral Higgs bosons could be light and close in mass.

6 One can rightfully use here the words of Winston Churchill in November 1942 after the battle of El Alamein (which in Arabic literally means “the two flags” but could also mean “the two worlds” or even “the two scientists”!): “Now, this is not the end; it is not even the beginning to the end; but it is, perhaps, the end of the beginning”.
